# cPLA_2_α targeting to exosomes connects nuclear deformation to LTB_4_-signaling during neutrophil chemotaxis

**DOI:** 10.1126/sciadv.aea2784

**Published:** 2026-02-20

**Authors:** Subhash B. Arya, Fatima Jordan-Javed, Kristen Loesel, Yehyun Choi, Samuel P. Collie, Lauren E. Hein, Brendon M. Baker, Euisik Yoon, Carole A. Parent

**Affiliations:** ^1^ Life Sciences Institute, University of Michigan, Ann Arbor, USA.; ^2^ Department of Cell and Developmental Biology, University of Michigan Medical School, Ann Arbor, USA.; ^3^ Cancer Biology Graduate Program, University of Michigan Medical School, Ann Arbor, USA.; ^4^ Department of Electrical Engineering and Computer Sciences, University of Michigan, Ann Arbor, USA.; ^5^ Cellular and Molecular Biology Graduate Program, University of Michigan Medical School, Ann Arbor, USA.; ^6^ Department of Biomedical Engineering, University of Michigan, Ann Arbor, USA.; ^7^ Department of Mechanical Engineering, University of Michigan, Ann Arbor, USA.; ^8^ Center for Nanomedicine, Institute for Basic Science, Yonsei University, Seoul, Republic of Korea.; ^9^ Department of Pharmacology, University of Michigan Medical School, Ann Arbor, USA.; ^10^ Rogel Cancer Center, University of Michigan Medical School, Ann Arbor, USA.

## Abstract

Efficient neutrophil chemotaxis requires the integration of mechanical forces and lipid-mediated signaling. While the signaling lipid leukotriene B4 (LTB_4_) reinforces cellular polarity, how mechanical cues regulate its production remains unclear. We now show that cytosolic phospholipase A2α (cPLA_2_α), which is essential for the synthesis of LTB_4_, functions as a nuclear curvosensor. cPLA_2_α responds to nuclear squeezing by localizing to ceramide-rich inner nuclear membrane microdomains and incorporating onto the exofacial surface of nuclear envelope–derived exosomes. This unique topology enables localized LTB_4_ synthesis, which synchronizes calcium spikes, promotes myosin light chain II phosphorylation, and sustains polarity and directional persistence after constriction. In neutrophils passing through tight spaces, cPLA_2_α activity drives the chemotactic response to nuclear squeezing by promoting exosomal LTB_4_ production and persistence after constriction. These findings uncover a cPLA_2_α-dependent mechanochemical axis linking nuclear architecture to chemotactic efficiency and offer alternative strategies to modulate inflammatory responses.

## Teaser

Nuclear squeezing triggers lipid signals that help neutrophils stay on course through tight spaces.

## INTRODUCTION

The four cardinal signs of inflammation are tumor (swelling), rubor (redness), dolor (pain), and calor (heat) (*1*). While redness, heat, and pain are mediated by chemicals such as histamine and prostaglandins, swelling results from the accumulation of fluids and immune cells in the affected tissue (*1*, *2*). Neutrophils are the first innate immune cells recruited to injured or infected sites in response to damage-associated molecular patterns, such as the chemoattractant N-formylmethionine-leucyl-phenylalanine (fMLF), in a process known as chemotaxis (*3*). In vivo, neutrophils engage in an actomyosin-driven, two-step “search and run” response to primary chemoattractants (*4*, *5*). This directional migration is amplified by neutrophil-derived leukotriene B4 (LTB_4_), a secondary chemoattractant that is essential for the rapid recruitment of neutrophils throughout injured tissues (*6*).

As the largest and stiffest organelle in the cell, the nucleus is a key regulator of inside-out signaling required for successful mechanotransduction—a process that converts mechanical input into biochemical signaling and gene expression changes (*7*). Changes in nuclear shape, size, and envelope composition influence how cells respond to mechanical forces, a process known as mechanosensing, through nuclear mechanotransduction. For example, depletion of the nucleoskeleton protein, lamin A/C (LMNA/C), promotes translocation of cytosolic phospholipase A2 alpha (cPLA_2_α) to the nuclear envelope (NE) upon swelling in HeLa cells and in response to hypotonic environments in zebrafish neutrophils (*8*). Moreover, in confined dendritic cells and zebrafish progenitor cells, cPLA_2_α activity promotes the redistribution of myosin II to the cell cortex, supporting migratory phenotypes (*9*–*11*). Although these results indicate that cPLA_2_α translocation depends on increased nuclear tension, the mechanosensitive role of the nucleus during chemotaxis in human neutrophils—particularly under constricted conditions that mimic tissue infiltration—remains poorly understood.

cPLA2α plays diverse roles in cellular physiology, ranging from initiating lipid signaling cascades to regulating membrane dynamics (*12*). Its primary enzymatic function is the selective hydrolysis of phospholipids at the sn-2 position, releasing arachidonic acid (AA)—the precursor of eicosanoids such as prostaglandins and leukotrienes—and lysophosphatidylcholine (LPC), a cone-shaped lipid that induces positive membrane curvature (*13*, *14*). While AA release is central to the biogenesis of inflammatory mediators, LPC production has been implicated in membrane remodeling and curvature generation. These dual outputs allow cPLA_2_α to operate at the intersection of signal transduction and membrane mechanics. In addition to its enzymatic activity, cPLA_2_α is itself a sensor and promoter of positive membrane curvature (*14*, *15*). Its N-terminal C2 domain facilitates calcium-dependent membrane binding and is sufficient to induce vesiculation both in vitro and in cells (*14*). This domain exhibits preferential localization to small, highly curved vesicles (~50 nm), while excluding flatter or larger structures (~600 nm) (*15*), underscoring its sensitivity to nanoscale curvatures. Such curvature-sensing features are particularly relevant in the nuclei of neutrophils, where extensive NE remodeling is required for the biogenesis of LTB_4_-producing exosomes (*16*). In this context, cPLA_2_α may serve a dual role—sensing extreme membrane curvature at budding NE domains and contributing enzymatically to the lipid composition and signaling of nascent exosomes.

AA release from membrane phospholipids is central to eicosanoid metabolism, serving as the precursor for both prostaglandins and leukotrienes. Although neutrophils express cyclooxygenases—the enzymes required for prostaglandin synthesis in lipid bodies—they typically contain few or no lipid droplets per cell (*17*) and produce minimal amounts of prostaglandins (*18*). As a result, leukotriene biosynthesis, particularly LTB_4_, predominates as the principal eicosanoid output in neutrophils. Upon chemoattractant stimulation, G protein–coupled receptor (GPCR) signaling induces a rise in intracellular calcium, which activates cPLA_2_α and releases AA (*19*). The endoplasmic reticulum (ER)/NE-resident, 5-lipoxygenase–activating protein (FLAP) then shuttles AA to 5-lipoxygenase (5LO), which sequentially converts it to 5-hydroxyeicosatetraenoic acid and leukotriene A_4_ (LTA_4_); LTA_4_ hydrolase (LTA_4_H) subsequently generates LTB_4_ (*20*). During chemotaxis, this entire LTB_4_-synthesizing ensemble—5LO, FLAP, and LTA_4_H—is loaded onto buds originating from neutral-sphingomyelinase (nSMase)–generated lipid microdomains on the NE (*21*). These NE buds mature into NE-derived multivesicular bodies (NE-MVBs) that fuse with the plasma membrane, releasing FLAP-positive, CD63-negative exosomes containing LTB_4_ (*16*, *22*). Extracellular LTB_4_ acts in an autocrine and paracrine fashion by binding to specific GPCRs to amplify the recruitment of additional neutrophils, sharpen the front-to-rear polarity of cells, and sustain high migratory persistence (*23*). Central to this amplification loop is the phosphorylation of myosin light chain II (MLC II), which reinforces rearward actomyosin contractility (*24*). In addition, LTB_4_ signaling simultaneously engages additional pathways that coordinate actin polymerization, integrin activation, and directional sensing, thereby ensuring robust collective chemotaxis (*25*).

Neutrophils respond to both chemical gradients and physical cues such as matrix stiffness, topography, and architecture (*26*). While components like integrins, TRPV/PIEZO channels, and the cytoskeleton have been implicated in mechanical responses, how mechanical and chemical signals are integrated in fast-migrating cells like neutrophils remains largely undefined (*27*). Notably, inhibition of cPLA_2_α in human neutrophils reduces LTB_4_ production and impairs transepithelial migration (*28*). Yet, the mechanistic underpinnings linking cPLA_2_α activity to exosome biogenesis and chemotaxis have remained elusive. In this study, we used a novel constricted chemotaxis chamber (C^3^), which introduces a single, physiologically relevant nuclear deformation during migration, to interrogate how mechanical stress on the nucleus regulates signaling outputs. We identify a mechanism by which nuclear squeezing promotes cPLA_2_α packaging onto the NE-derived exosomes, and subsequent LTB_4_-dependent reinforcement of neutrophil chemotaxis.

## RESULTS

### Neutrophil nuclear morphology is regulated by cPLA_2_α

**Fig. 1.**
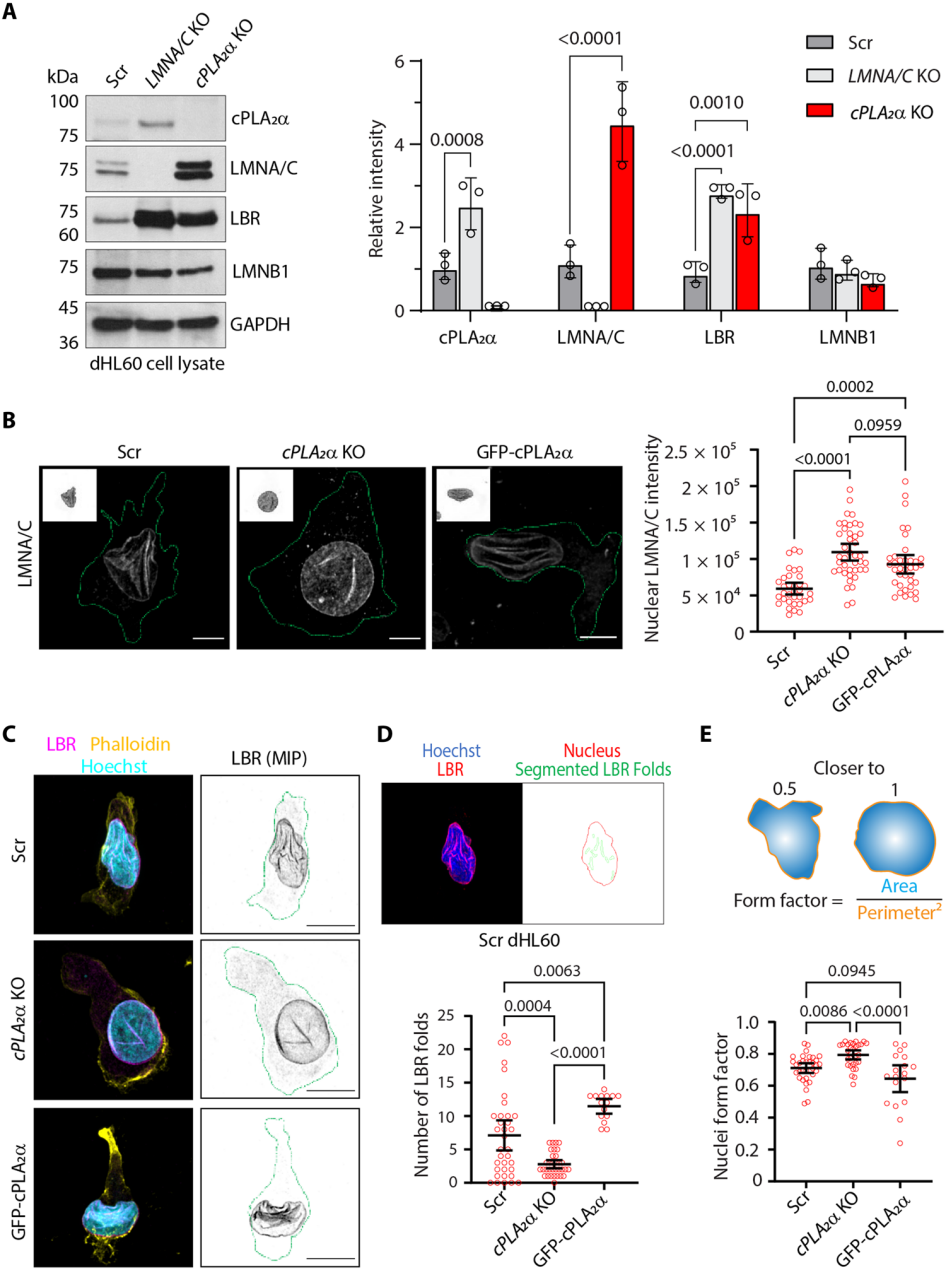
Effect of cPLA_2_α on nuclear morphology in dHL60 neutrophils. (**A**) Representative immunoblots of dHL60 neutrophils, along with the respective quantification graph showing the level of indicated proteins in the cell lysates. Data are presented as means ± SD and *P* values calculated using two-way analysis of variance (ANOVA). *N* = 3. (**B**) Representative airyscan microscopy images of fixed dHL60 neutrophils chemotaxing underagarose showing the nuclear levels of LMNA/C quantified and presented as a scatter plot with mean ± 95% confidence interval (CI) of >30 data points (circles) pooled from three independent experiments. Scale bar, 5 μm; the green line represents the cell boundary. *P* values determined using ordinary one-way ANOVA are shown. (**C**) Representative airyscan microscopy images of dHL60 neutrophils migrating toward fMLF under agarose, fixed, and immunostained for LBR (magenta), phalloidin (yellow), and Hoechst (blue). Scale bar, 5 μm; the green line represents the cell boundary. (**D** and **E**) Scatter plots showing the effect of cPLA_2_α on the number of NE folds and nuclei form factor, presented as mean ± 95% CI of >30 data points (circles) pooled from three independent experiments. The top panels show nuclear outlines and LBR folds (D) as segmented using CellProfiler, and nuclear morphology described by form factor (E). *P* values determined using ordinary one-way ANOVA are shown.

To directly assess the role of cPLA_2_α in nuclear mechanotransduction during neutrophil chemotaxis, we used human promyelocytic leukemia–derived HL60 cells, which can be readily differentiated into neutrophil-like cells in the presence of dimethyl sulfoxide (DMSO) (*29*), and generated *cPLA_2_*α knockout (KO) cells using CRISPR-Cas9 (fig. S1A). To assess cPLA_2_α-specific effects, we exogenously expressed green fluorescent protein (GFP)–cPLA_2_α in *cPLA_2_*α KO (fig. S1B). Surface expression of CD11b levels on differentiated HL60 (dHL60) neutrophils confirmed comparable levels of differentiation across all cell lines (fig. S1C). Using anti-cPLA_2_α and anti-GFP antibodies, we confirmed that GFP-cPLA_2_α exhibits the same nucleocytosolic distribution as endogenous cPLA_2_α in undifferentiated HL60 cells. The anti-GFP antibody strongly labeled the overexpressed fusion protein, and the anti-cPLA_2_α signal was abolished in *cPLA_2_*α KO cells, confirming antibody specificity (fig. S1D). We next measured the expression and localization of the nuclear structural proteins, LMNA/C and lamin B receptor (LBR), both implicated in neutrophil nuclear lobulation (*30*). We found that *cPLA_2_*α KO and *LMNA/C* KO dHL60 cells express elevated levels of LBR. In contrast, while *cPLA_2_*α KO dHL60 neutrophils exhibit a significant up-regulation of LMNA/C, *LMNA/C* KO dHL60 cells have elevated cPLA_2_α expression compared to Scr dHL60 neutrophils. The expression of lamin B1 (LMNB1) remains unchanged (Fig. 1A). Elevated nuclear LMNA/C levels in the *cPLA_2_*α KO dHL60 cells were confirmed by immunofluorescence microscopy of cells chemotaxing under agarose (Fig. 1B).

Although dHL60 neutrophils lack distinct nuclear lobes observed in human polymorphonuclear neutrophils (PMNs), they display a complex, folded nuclear morphology characterized by multiple NE invaginations/folds enriched with the inner nuclear membrane (INM)–resident protein LBR (Fig. 1C, Scr cells) (*22*, *30*). In contrast, *cPLA_2_*α KO cells exhibit a rounder and less-folded nuclear morphology (Fig. 1C). Quantification of LBR-positive NE folds/invaginations and nuclear surface complexity (form factor)—measured using CellProfiler-based object segmentation—revealed a reduction in LBR-positive invaginations and an increase in nuclear form factor in the KO cells relative to the Scr controls (Fig. 1, D and E). Notably, reexpression of GFP-cPLA_2_α in KO cells restores the nuclear lobulation and surface complexity to levels comparable to Scr cells (Fig. 1, C to E, and movie S1). Together, these findings demonstrate that cPLA_2_α regulates LMNA/C expression and maintains the characteristic nuclear morphology of neutrophils.

### Neutrophils lacking cPLA_2_α exhibit defective nuclear mechanosensitivity

**Fig. 2.**
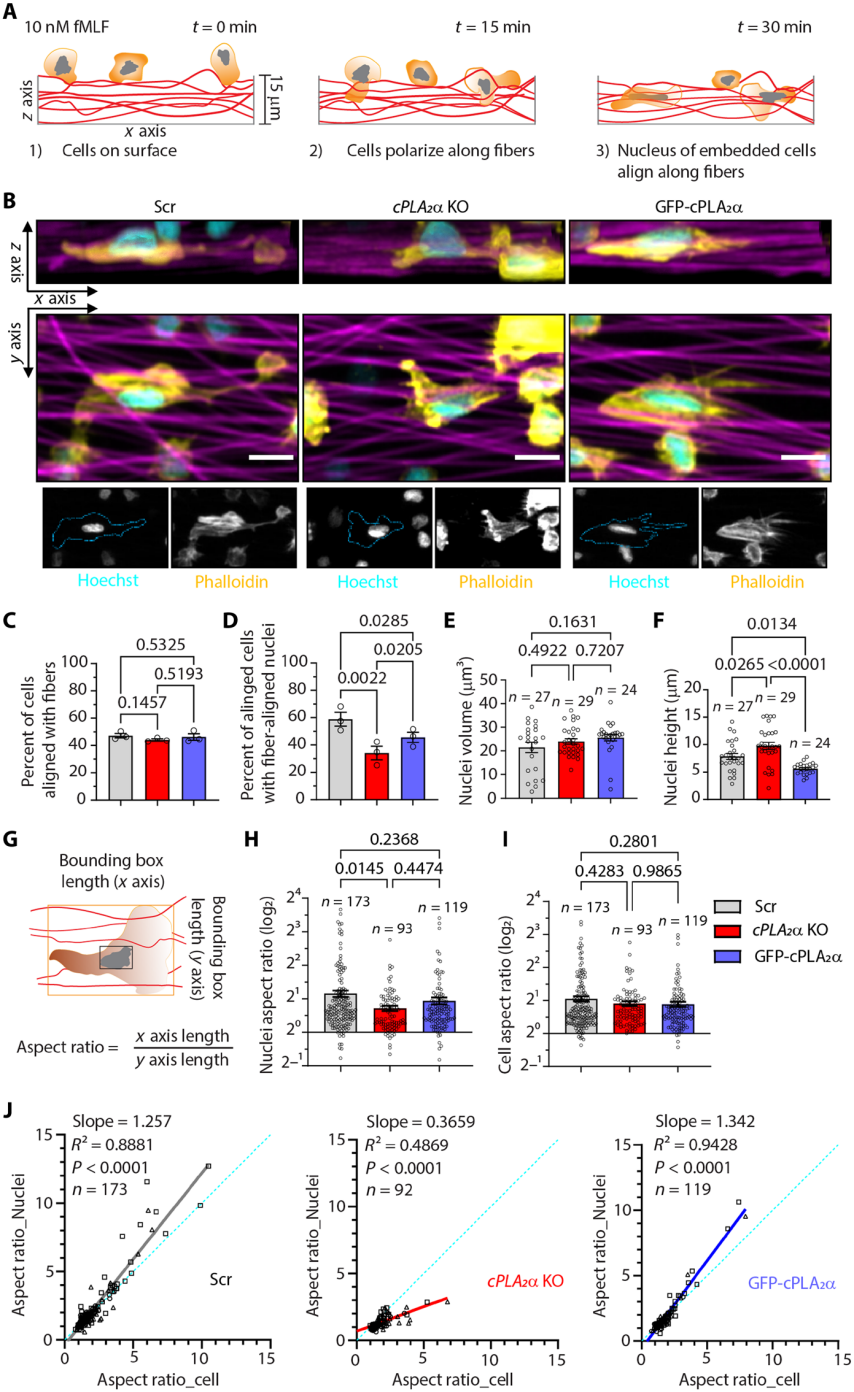
Effect of cPLA_2_α on cellular mechanosensing and nuclear mechanotransduction in activated dHL60 neutrophils. (**A**) Schematic depicting the behavior of neutrophils and their nuclei when plated on sADF fibslips in the presence of fMLF. (**B**) Representative confocal microscopy images of dHL60 neutrophils showing the shape of cells (phalloidin, yellow) and their nuclei (Hoechst, cyan) on aligned fibers (magenta), along the *xy* and *xz* axes, 30 min post–fMLF treatment. Scale bar, 5 μm. (**C** and **D**) Graphs plotted as means ± SEM showing the percent of cells aligned with fibers (C) and the percent of aligned cells with fiber-aligned nuclei (D). *N* = 3. (**E** and **F**) Graphs plotted as means ± SEM of data points pooled from three independent experiments, showing the nuclear volume (E) and the height of nuclei (F) within all aligned cells. (**G**) The approach used to calculate the cell and nuclei aspect ratio. The orange outline denotes the cell bounding box, and the black outline denotes the nuclear bounding box. Wavy red lines denote aligned microfibers. (**H** and **I**) Graphs plotted as means ± SEM of data points pooled from three independent experiments, showing the changes in the nuclear (H) and cellular (I) aspect ratio in Scr, *cPLA_2_*α KO, and GFP-cPLA_2_α cells. *P* values determined using repeated measures (RM) one-way ANOVA (C and D) and ordinary one-way ANOVA (E and F and H and I) are shown. (**J**) Graphs showing the correlation of cell and nuclei aspect ratio in dHL60 neutrophils plated on aligned fibers and activated with fMLF for 30 min.

Since both the LMNA/C (*31*) and cPLA_2_α (*8*, *9*) are involved in regulating cellular and nuclear mechanosensing in cancer cells, necrotic epithelial cells, and dendritic cells, we next evaluated the role of cPLA_2_α on cellular mechanosensing and nuclear mechanotransduction in migrating neutrophils. Efficient nuclear mechanotransduction is reflected by proportional changes in nuclear shape relative to cell shape in response to mechanical cues (*32*). To measure these parameters at the single-cell level, we plated fMLF-activated dHL60 neutrophils onto glass coverslips (fibslips) coated with fibrinogen-functionalized synthetic aligned dense fibers (sADFs), composed of cell-inert polymer dextran vinyl sulfone (DexVS), fabricated by electrospinning (*33*). We then analyzed cellular and nuclear morphology along the direction of fiber alignment. Within 15 min of plating, we observed that Scr and GFP-cPLA_2_α cells align with the sADF, and by 30 min, their nuclei are also aligned and embedded within the ~15-μm-thick sADF mats. While approximately half of all cell types aligned with the sADF within 30 min (Fig. 2, A to C, G, and I), significantly fewer *cPLA_2_*α KO neutrophils oriented their nuclei with the fiber direction or intercalated within fibers compared to Scr or GFP-cPLA_2_α cells (Fig. 2, B and D). Although nuclear volume was comparable across all cell lines (Fig. 2E), *cPLA_2_*α KO cells exhibited increased nuclear height with a concomitant decline in nuclear elongation relative to Scr and GFP-cPLA_2_α neutrophils within sADF-aligned populations (Fig. 2, F to H, and movie S2). Consistent with this, we observed a marked decrease in the correlation between cellular and nuclear elongation in fMLF-activated *cPLA_2_*α KO neutrophils compared to Scr and GFP-cPLA_2_α neutrophils on sADF (Fig. 2J). These findings suggest that in *cPLA_2_*α KO neutrophils, increased nuclear stiffness—driven by elevated LMNA/C expression—impairs the dynamic remodeling of nuclear membrane curvature required for nuclear entry and elongation within the channel-like architecture of dense, aligned fiber mats.

### Nuclear squeezing promotes cPLA_2_α-dependent increases in directionality during neutrophil chemotaxis

**Fig. 3.**
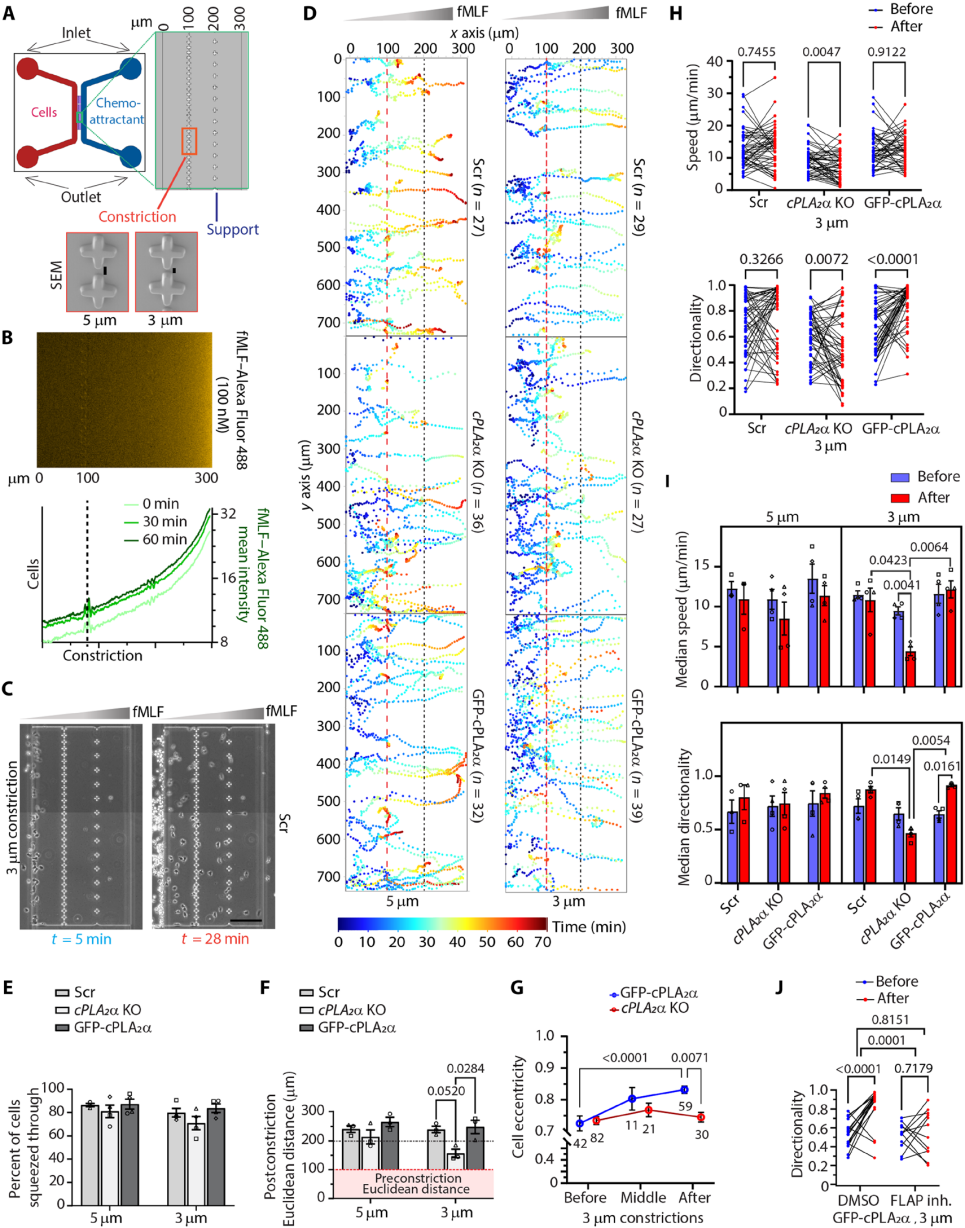
Effect of cPLA_2_α on dHL60 neutrophils chemotaxing through transient constrictions. (**A**) Image of the C^3^ showing the cell and chemoattractant inlet/outlet and the migration chamber. The zoomed inset (green) shows the migration chamber, and the zoomed insets orange) show SEM images of the 3- and 5-μm constrictions. (**B**) Image (top) and graph (bottom) showing the diffusion rate and gradient stability of fMLF–Alexa Flour 488 in C^3^. (**C**) Phase contrast images of Scr dHL60 neutrophils migrating toward fMLF through 3-μm constrictions in C^3^ at different time points. Scale bar, 100 μm. (**D**) Color-coded tracks of individual cells migrating toward the fMLF, through either 5- or 3-μm constrictions. Refer to the temporal color map on the right. The red dashed line indicates the point of constriction 100 μm from the migration start site. The black dashed line indicates the support site. (**E** and **F**) Graph showing the percentage of cells entering the C^3^ that squeezed through 5- or 3-μm constrictions (E) and the postconstriction Euclidean distance (F). *N* = 3. (**G**) Graph plotted as means ± SEM showing the change in cell eccentricity in response to constrictions during chemotaxis. *N* = 3. (**H**) Before-after graph showing the change in speed and directionality of chemotaxing cells post–3-μm constrictions. Randomized data points (*50*) from three independent experiments are plotted. The *P* values calculated using multiple paired *t* test are presented. (**I**) Graphs showing the changes in postconstriction median speed and median directionality of chemotaxing Scr, *cPLA_2_*α KO, and GFP-cPLA_2_α dHL60 cell populations, compared to preconstriction parameters. *N* ≥ 3. Graphs are plotted as means ± SEM. The *P* values determined using three-way ANOVA are shown. (**J**) Before-after graph showing the change in the directionality of chemotaxing GFP-cPLA_2_α expressing cells treated with either DMSO or FLAP inhibitor (MK886) in 3-μm constrictions. *P* values determined using two-way ANOVA are shown.

To investigate how neutrophils specifically respond to nuclear constrictions during chemotaxis, we developed the C^3^, a microfluidic device designed to expose chemotaxing cells to a single, transient constriction. The C^3^ device is composed of polydimethylsiloxane (PDMS) bonded to a glass coverslip via plasma activation and features diametrically opposed inlets and outlets for cell and chemoattractant loading, each 100 μm high (Fig. 3A). The migration channels are 5 μm in height, 300 μm in length, and contain mechanical confinement pillars of various gap sizes positioned 100 μm from the cell inlet (Fig. 3A, zoomed inset). We selected a cross-shaped constriction pillar (20 μm across) over conventional cylindrical posts (*34*, *35*), as the cross geometry produced higher curvature constrictions suited for our assay, whereas cylindrical columns frequently resulted in undesired cell wrapping during migration. This design enables transient entrapment of migrating neutrophils in a cup-like space between two constriction pillars, compelling them to squeeze through defined constriction gaps (Fig. 3A, zoomed inset). Given the average nuclear diameter of dHL60 neutrophils (~4 μm; Fig. 2E), we selected constriction widths of 5 and 3 μm to represent conditions of unrestricted and restricted nuclear passage and nuclear squeezing, respectively. Last, using Alexa Fluor 488–tagged fMLF, we verified that the chemoattractant gradient establishes within 15 min and remains stable over 60 min without external flow control (Fig. 3B). Thus, the C^3^ device allows for the direct visualization and quantification of nuclear squeezing and how this impacts neutrophil chemotaxis (Fig. 3C).

Analysis of Hoechst-stained dHL60 neutrophils revealed that at least 80% of Scr, GFP-cPLA_2_α, and, unexpectedly, *cPLA_2_*α KO neutrophils successfully migrate through 3- and 5-μm constrictions within 1 hour (Fig. 3, D and E). Using the ImageJ “TrackMate” plugin for cell tracking and MATLAB for speed and directionality analysis (see Materials and Methods), we found that most Scr and GFP-cPLA_2_α dHL60 neutrophils migrate ~150 μm beyond the constriction, regardless of constriction width (Fig. 3, D and F). We reason that the cell arrest near 250 μm from the inlet likely reflects saturating chemoattractant levels near the fMLF outlet (300 μm from the cell inlet). While *cPLA_2_*α KO cells perform similarly to Scr and GFP-cPLA_2_α cells in the 5-μm C^3^ device, they migrate significantly shorter distances after traversing 3-μm constrictions (Fig. 3, D and F, and movie S3). This postconstriction impairment correlates with loss of cell polarity in *cPLA_2_*α KO cells, in contrast to the enhanced polarity observed in GFP-cPLA_2_α cells (Fig. 3G). Moreover, unlike Scr and GFP-cPLA_2_α neutrophils, *cPLA_2_*α KO cells reduce their speed after 3-μm constrictions and exhibit reduced directionality (Fig. 3, H and I). Notably, GFP-cPLA_2_α cells, which express elevated cPLA_2_α levels compared to Scr controls (fig. S1B), show a significant postconstriction increase in directionality (Fig. 3, H and I). Whereas all cell types slow down when passing through 5-μm constrictions as they approach saturating chemoattractant, nuclear squeezing through 3-μm constrictions maintains speed in a cPLA_2_α-dependent manner (Fig. 3I). We also found that a higher fraction of chemotaxing Scr dHL60 cells exhibit increased directionality after squeezing through 3-μm, but not 5-μm, constrictions (fig. S2, A and B). In contrast, this subpopulation shift was absent in *cPLA_2_*α KO neutrophils and more pronounced in cells expressing elevated cPLA_2_α levels (i.e., GFP-cPLA_2_α dHL60 neutrophils) chemotaxing through 3-μm constrictions (fig. S2B). To determine whether this response depends on leukotriene signaling, we pretreated GFP-cPLA_2_α cells with MK886, an FLAP inhibitor that blocks LTB_4_ production (*36*). Strikingly, MK886 treatment abolished the postconstriction increase in directionality observed in untreated GFP-cPLA_2_α cells (Fig. 3J). To determine the impact of LMNA/C expression on the ability of cPLA_2_α to modulate neutrophil chemotactic performance in response to nuclear squeezing, we used *LMNA/C* KO dHL60 cells and PMNs, which naturally lack LMNA/C (*30*), and the cPLA_2_α inhibitor MAFP (*37*). We found that both cell types show similar cPLA_2_α-dependent increases in directionality after crossing 3-μm constrictions (fig. S2, C and D). Inhibition of cPLA_2_α in either *LMNA/C* KO neutrophils or human PMNs also led to a decrease in cell speed post–3-μm constrictions, which is maintained in DMSO-treated cells (fig. S2, C and D). These results were recapitulated in PMNs treated with MK886 (fig. S2D). Together, these findings show that irrespective of the presence of LMNA/C, cPLA_2_α levels regulate dHL60 neutrophil and PMN chemotaxis in response to nuclear squeezing, with higher expression promoting directional persistence in an LTB_4_-dependent manner.

**Fig. 4.**
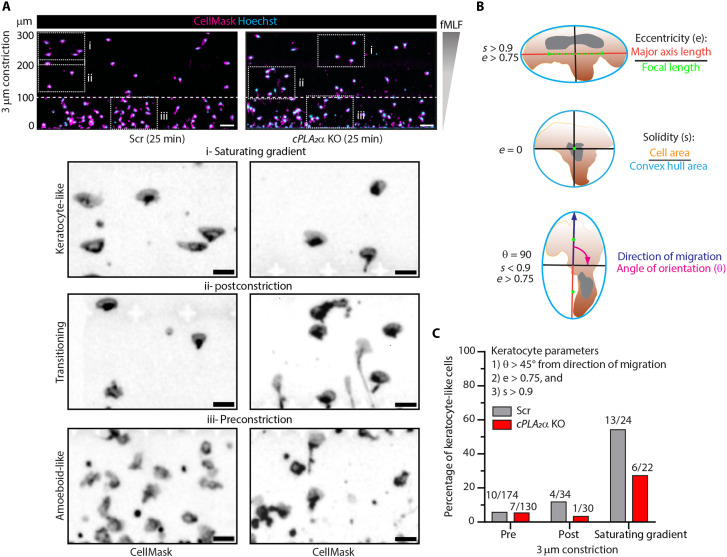
Loss of cPLA_2_α inhibits amoeboid-to-keratocyte–like migratory transition in response to nuclear squeezing. (**A**) Representative microscopy images of dHL60 cells stained with CellMask (PM, magenta) and Hoechst 33342 (nucleus, cyan) 25 min after the start of chemotaxis in 3-μm C^3^ devices, showing the morphological transitions from amoeboid-like to keratocyte-like migration mode (see insets). The dashed white line indicates the position of constrictions. Scale bar, 10 μm, and 20 μm in the zoomed insets. *N* = 2. (**B**) Schematic showing the metrics used for measuring various cellular parameters. (**C**) Graph showing the percentage of keratocytes in Scr versus *cPLA_2_*α KO cells, and the classifier used to ascertain keratocyte-like morphology.

While Scr dHL60 neutrophils initially migrate in an amoeboid pattern through the initial one-third of the C^3^ chamber (before the constrictions), we found that they transition into a fan-shaped, keratocyte-like morphology as they approach higher fMLF concentrations—particularly beyond the 3-μm constrictions (Fig. 4A, top). Keratocyte-like cells, defined by a morphology showing a major axis perpendicular to the direction of migration, exhibit a smoother cell membrane compared to amoeboid cells (*38*, *39*). On the basis of this information, we used CellProfiler-based object segmentation (Fig. 4B) to classify migrating neutrophils as keratocyte-like if their orientation exceeded 45°, eccentricity >0.75, and solidity >0.9. Notably, we found that cPLA_2_α depletion reduces this amoeboid-to-keratocyte–like transition by nearly 50% (Fig. 4, A and C). Accompanying this defect, we observed persistent uropod extension in *cPLA_2_*α KO cells that remained polarized postconstriction, while most others lost polarity (movie S3). Together, these results indicate that cPLA_2_α is critical for the morphological and migratory transitions required for directional persistence in neutrophils undergoing nuclear squeezing.

### Nuclear squeezing drives cPLA_2_α-dependent calcium spikes and MLC II phosphorylation during neutrophil chemotaxis

**Fig. 5.**
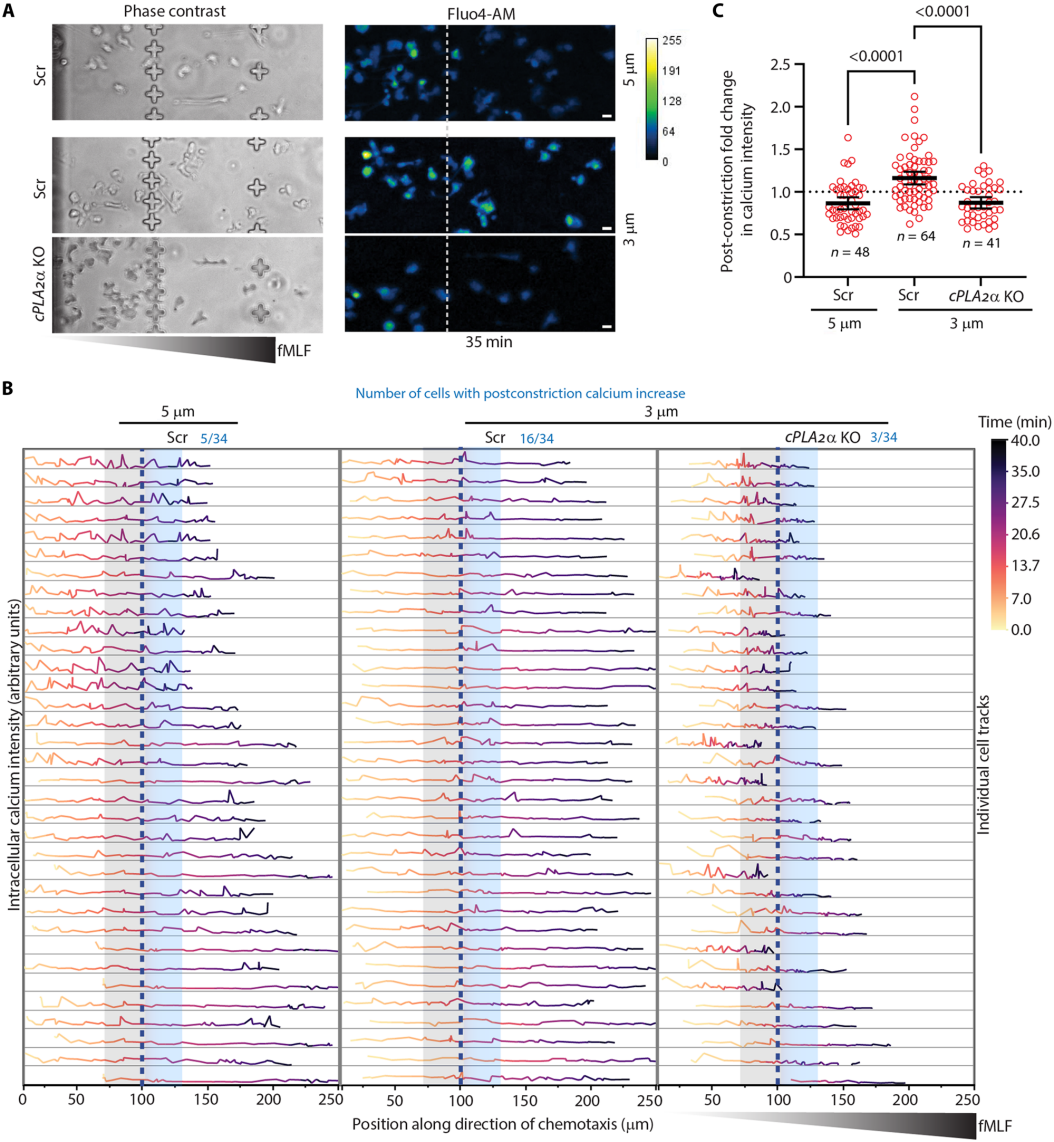
Calcium dynamics in chemotaxing neutrophils in response to nuclear squeezing. (**A**) Representative microscopy images of Fluo4-AM–labeled Scr and *cPLA_2_*α KO dHL60 neutrophils chemotaxing toward fMLF and traversing either 5- or 3-μm constrictions in C^3^ devices. Dashed white line marks the constriction site. Pseudocolor scale (blue-green-yellow) reflects increasing intracellular calcium intensity. Scale bar, 5 μm. (**B**) Single-cell traces of calcium intensity aligned along the direction of chemotaxis. Calcium intensity (*y* axis) is plotted against distance traveled (*x* axis), with trace color indicating time (yellow, earlier; blue, later). The vertical blue line denotes the constriction position, and gray-blue–shaded regions denote one cell length (25 μm) used to quantify the pre- and postconstriction calcium intensity plotted in (C). (**C**) Scatter plot showing the fold change in postconstriction Fluo4-AM intensity for individual cells, presented as means ± 95% CI. The *P* values were calculated using ordinary one-way ANOVA; pooled cell numbers from two independent experiments are indicated as *n*.

The catalytic activity and membrane recruitment of cPLA_2_α require elevated intracellular calcium, which can be induced by GPCR signaling (*19*, *40*, *41*). Similarly, 5LO can be activated by calcium fluxes required for LTB_4_ synthesis (*42*). LTB_4_ signaling via GPCR activation leads to increases in intracellular calcium and MLC II phosphorylation, processes critical for stable uropod formation and persistent migration (*24*, *43*). Notably, neutrophils migrating under agarose via an adhesion-independent “chimneying” mode depend heavily on actomyosin contractility (*44*, *45*). To investigate how cPLA_2_α regulates calcium signaling in response to nuclear squeezing, we analyzed both single-cell calcium dynamics and population-level responses in neutrophils chemotaxing toward fMLF in C^3^ devices using Fluo4-AM staining (Fig. 5A) (*46*). Single-cell calcium tracing revealed that nuclear squeezing elicits a synchronized response, with ~50% Scr cells displaying prominent calcium spikes immediately after 3-μm constrictions (Fig. 5B, middle), whereas post–5-μm constrictions, subpopulations of Scr neutrophils exhibit spontaneous, asynchronous calcium spikes as they approach saturating fMLF gradients (Fig. 5B, left). In contrast, *cPLA_2_*α KO neutrophils lack postconstriction calcium spikes as they squeeze through 3-μm constrictions, consistent with the loss of Euclidean distance and polarity (Figs. 5B and 3, F and G). At a population level, we found that postconstriction calcium intensity is markedly higher in Scr neutrophils squeezing through 3-μm, compared to 5-μm constrictions (Fig. 5, A to C). In contrast, *cPLA_2_*α KO neutrophils lack this postconstriction increase in calcium spikes (Fig. 5C). These results suggest a role for cPLA_2_α-dependent calcium signaling in actomyosin regulation, which is involved in maintaining cell polarity.

**Fig. 6.**
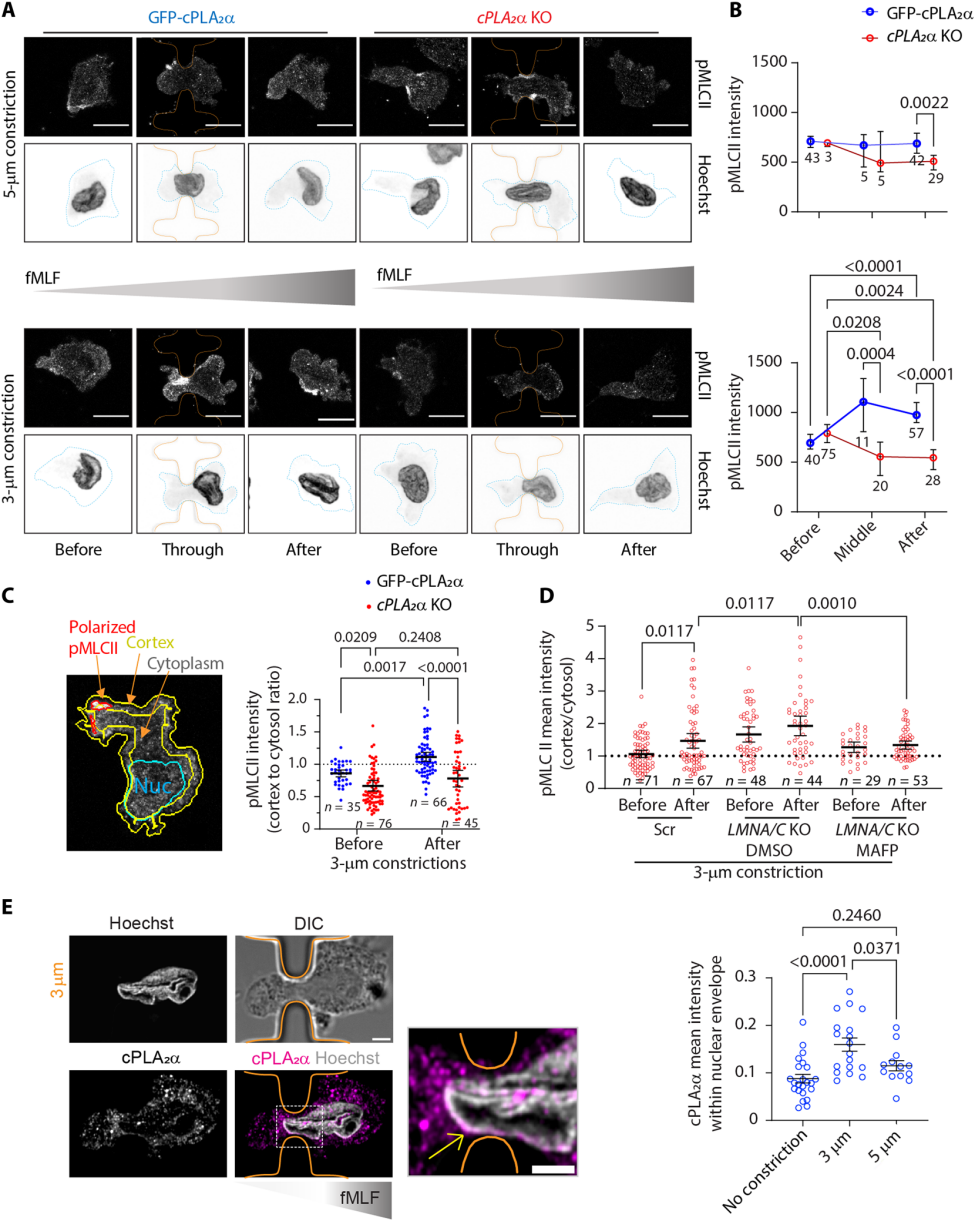
Effect of cPLA_2_α on postconstriction MLC II phosphorylation and cell polarization in dHL60 neutrophils. (**A**) Representative confocal microscopy images of dHL60 neutrophils chemotaxing toward fMLF, fixed, and immunostained for pMLC II and Hoechst 33342 (nuclei). Blue outline indicates the cell periphery, and orange outline indicates constriction pillars. Scale bar, 10 μm. (**B**) Graphs plotted as means ± SEM, showing the change in total pMLC II intensity within the cell before, during, and after constrictions. *N* = 3. (**C**) Representative microscopy image (left) showing the cortex (yellow), nucleus (cyan), and polarized pMLC II (red) outlines. The scatter dot plot (right) shows the cortex-to-cytosol pMLC II intensity ratio in the chemotaxing cells before and after 3-μm constrictions. *P* values determined using two-way ANOVA are shown. The number of data points (*n*) pooled from three independent experiments are plotted as means ± 95% CI are mentioned on the graph. (**D**) Scatter dot plot showing the cortex-to-cytosol pMLC II intensity ratio in chemotaxing cells before and after 3-μm constrictions, presented as means ± 95% CI. *P* values determined using ordinary one-way ANOVA are shown, and the number of data points (*n*) pooled from two independent experiments is mentioned. (**E**) Airyscan microscopy images (left) of a GFP-cPLA_2_α dHL60 neutrophil squeezing through a 3-μm constriction, immunostained for cPLA_2_α (magenta) and Hoechst (gray), along with the phase contrast image and individual channels in grayscale. Orange outlines the constriction pillars, and the yellow arrow points to the NE enrichment of cPLA_2_α at the constriction. Scale bar, 3 μm, and 2 μm in the zoomed inset. Scatter dot plot (right) showing the changes in cPLA_2_α levels at the NE before and after 3- and 5-μm constrictions. Data points (circles) from three independent experiments were pooled and plotted as means ± 95% CI, and *P* values determined using two-way ANOVA are presented.

Persistent migration requires robust actomyosin contractility, particularly at the cell rear (*4*). We previously demonstrated that inhibition of LTB_4_ biosynthesis in fMLF-stimulated neutrophils markedly reduces phosphorylation of MLC II and impairs chemotaxis (*23*, *24*). Given that migration through 3-μm constrictions induces a morphological transition from an amoeboid- to a keratocyte-like phenotype (Fig. 4) and enhances directional persistence (Fig. 3, H and I), we next investigated whether this transition involves changes in myosin activity and its cortical redistribution. Using object-based segmentation, we quantified levels and localization of phosphorylated MLC II (pMLC II) in *cPLA_2_*α KO and GFP-cPLA_2_α neutrophils before, during, and after passage through 5- and 3-μm constrictions. We observed a significant increase in intracellular pMLC II intensity specifically in GFP-cPLA_2_α cells during and after transit through 3-μm constrictions (Fig. 6, A and B). Moreover, the cortex-to-cytosol pMLC II ratio increased exclusively in GFP-cPLA_2_α cells navigating through 3-μm constrictions (Fig. 6C), coinciding with enhanced cell polarity (Fig. 3G). This effect was recapitulated in *LMNA/C* KO neutrophils treated with MAFP (Fig. 6D). Notably, *LMNA/C* KO neutrophils exhibit a heightened pMLC II polarization, compared to Scr cells, suggesting that increased expression of cPLA_2_α correlates with pMLC II polarity (Figs. 6D and 1A). These findings indicate that cPLA_2_α activity promotes actomyosin contractility in response to nuclear squeezing, enabling persistent chemotaxis. We envision that nuclear squeezing activates cPLA_2_α, triggering LTB_4_ production, calcium influx, and downstream MLC II phosphorylation.

**Fig. 7.**
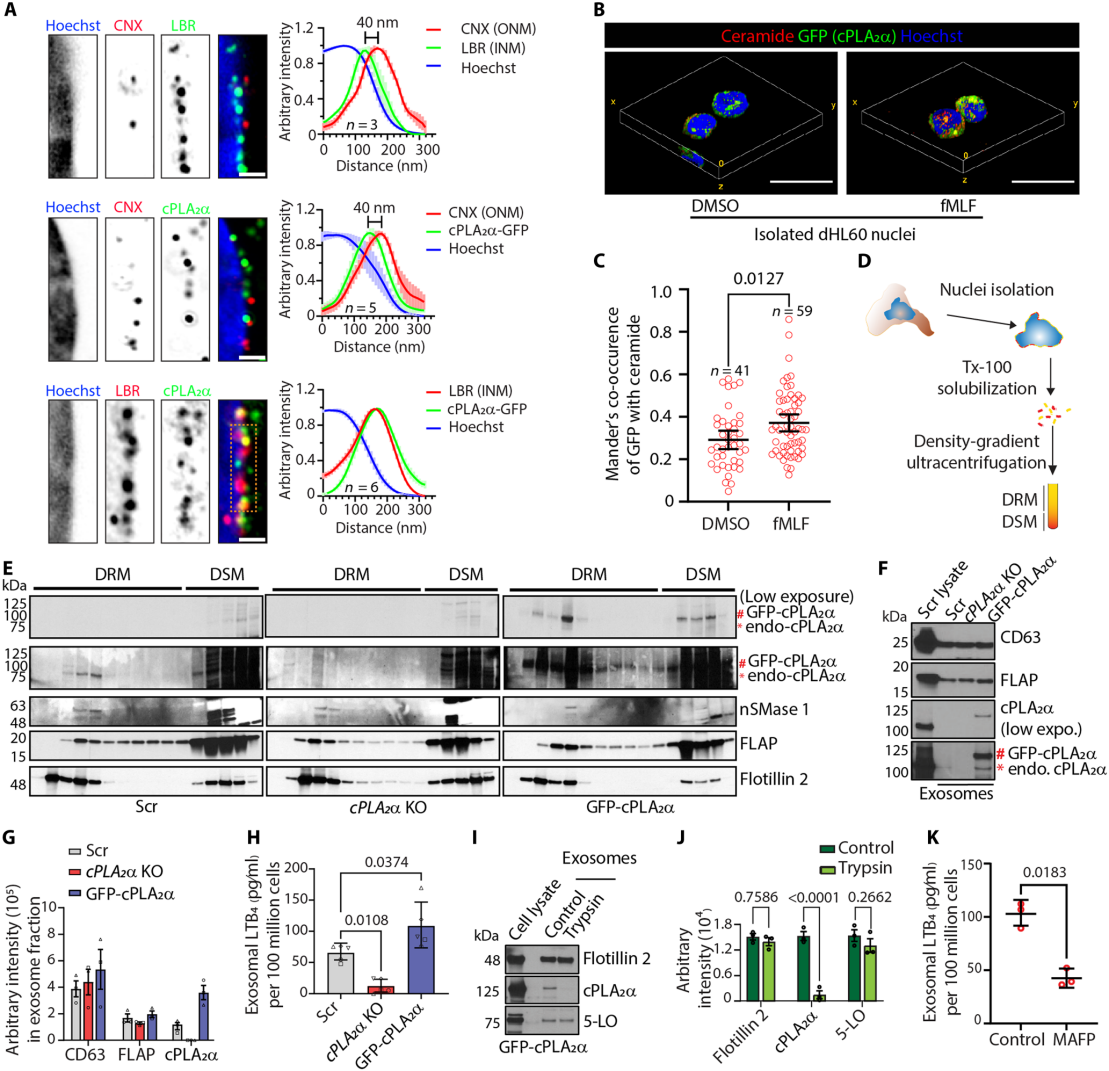
cPLA_2_α is enriched at INM microdomains and on the exofacial surface of exosomes in activated dHL60 neutrophils. (**A**) Fourfold expansion microscopy images of chemotaxing GFP-cPLA_2_α dHL60 cells and immunostained with the indicated antibodies. Orange dashed outlines mark regions used for fluorescence intensity profiling. Histograms show fluorescence intensity profiles averaged across multiple images (*n*) and plotted as means ± SEM. Solid lines represent the mean, and shaded bars indicate the standard error. *N* = 3. Scale bar, 300 nm. (**B** and **C**) Three-dimensional volumetric view (B) of fixed and immunostained nuclei isolated from dHL60 neutrophils showing the distribution of GFP-cPLA_2_α (green), ceramide (red), and Hoechst 33342 (blue), quantified and presented as the scatter plot (C) showing Mander’s co-occurrence. Data points (*n*) are plotted as means ± 95% CI. *N* = 3. Scale bar, 10 μm. (**D** and **E**) Schematic (D) depicting the steps involved in the isolation of NE-membrane microdomains, and immunoblots (E) showing the cPLA_2_α, FLAP, nSMase1, and Flotillin 2 distribution in DRM and DSM fractions of NE obtained from activated cells. *N* = 4. (**F** and **G**) Representative immunoblot (F) and graph (G) plotted as means ± SD, showing the levels of CD63, FLAP, and cPLA_2_α in purified exosomes obtained from various cell lines. *N* = 3. (**H**) Graph showing the levels of LTB_4_ within the exosomes purified from various cell lines upon fMLF activation. *N* = 5. (**I** and **J**) Representative immunoblots (I) and graph (J) plotted as means ± SD, showing the levels of cPLA_2_α, 5LO, and Flotillin 2 in the purified exosomes upon trypsin treatment. *N* = 3. (**K**) Scatter dot plot showing the levels of LTB_4_ within exosomes isolated from fMLF-activated PMNs and treated in vitro either with DMSO or MAFP for 30 min at 37°C. Data points (red circles) representing three independent experiments are plotted as means ± SD. The *P* values determined using Mann-Whitney test (C), two-way ANOVA (G and J), and Student *t* test (K) are presented.

Zebrafish cPLA_2_α, a predominantly nuclear-localized protein, has been shown to translocate from the nucleus to the NE (*8*, *47*). Consistent with previously reported nucleo-cytosolic distribution of cPLA_2_α (*19*, *48*), we found that in chemotaxing GFP-cPLA_2_α neutrophils, ~30% of the GFP-cPLA_2_α signal is nuclear, with ~20% localized to the nucleoplasm and ~10% enriched at the NE (fig. S3, A and B). Notably, GFP-cPLA_2_α further accumulates at the NE in neutrophils migrating through 3-μm (but not 5-μm) constrictions, coinciding with increased pMLC II levels (Fig. 6E). Using 4× expansion microscopy to enhance NE resolution, we found that in chemotaxing neutrophils, GFP-cPLA_2_α localizes to LBR-positive INM, but not to calnexin-positive outer nuclear membranes (Fig. 7A). These results suggest that nuclear squeezing promotes the recruitment of cPLA_2_α to the INM.

### cPLA_2_α is recruited to the exofacial surface of NE-derived exosomes

We previously showed that neutrophils undergoing chemotaxis exhibit NE budding at ceramide-rich, lipid-ordered membrane microdomains, which requires nSMase activity on the NE (*16*). We now find that in dHL60 neutrophils, GFP-cPLA_2_α colocalizes with ceramide-rich membrane microdomains on the NE of isolated nuclei, and Mander’s coefficient analysis reveals increased colocalization upon fMLF stimulation, indicating activation-dependent redistribution (Fig. 7, B and C, and movie S4). In addition, both endogenous and GFP-tagged cPLA_2_α are found in lipid-ordered, detergent-resistant membrane (DRM) fractions isolated from the NE of fMLF-stimulated cells (Fig. 7, D and E, and fig. S3C). We found that cPLA_2_α is not required for ceramide-rich membrane microdomain formation, as the absence of cPLA_2_α does not alter the enrichment of Flotillin 2, FLAP, or nSMase 1 in these domains (Fig. 7E and fig. S3C). Thus, while cPLA_2_α localizes to these lipid microdomains, it is dispensable for their assembly in neutrophils.

Because the LTB_4_ synthesis machinery—5LO, FLAP, and LTA_4_H—is present in NE-derived LTB_4_-containing exosomes (*16*, *21*), we asked whether cPLA_2_α is also in these vesicles. Density-gradient ultracentrifugation of fMLF-stimulated dHL60 neutrophil supernatants revealed that cPLA_2_α is also present in purified exosomes containing 5LO/FLAP (Fig. 7, F and G). Although the absence of cPLA_2_α did not affect the recruitment of 5LO or FLAP to exosomes, as expected, LTB_4_ levels were completely abolished in exosomes isolated from *cPLA_2_*α KO cells (Fig. 7H). Conversely, GFP–cPLA_2_α neutrophils had elevated exosomal LTB_4_ levels compared to Scr controls, consistent with their higher cPLA_2_α expression (Fig. 7, F to H).

Given the ability of cPLA_2_α to bind positively curved membranes and the high curvature of the exofacial leaflet of exosomes, we reasoned that cPLA_2_α is present on the outer surface of exosomes. To test this, we treated exosomes purified from fMLF-stimulated GFP-cPLA_2_α dHL60 neutrophils with trypsin. As expected, trypsin does not affect the level of intra-exosomal proteins like Flotillin 2 (*49*) and 5LO but completely abolishes the GFP-cPLA_2_α signal (Fig. 7, I and J), showing that cPLA_2_α is exposed on the exofacial surface of NE-derived exosomes. Notably, we also found that exosomes isolated from fMLF-stimulated PMNs, and subsequently treated with MAFP, have significantly reduced LTB_4_ levels compared to DMSO controls (Fig. 7K), showing that exosomal cPLA_2_α is enzymatically active. Collectively, these findings support a model in which nuclear squeezing generates extreme positive curvature at the INM, forming hotspots for cPLA_2_α enrichment near NE-budding sites. This membrane-associated pool of cPLA_2_α becomes incorporated onto the outer surface of highly curved intraluminal vesicles, which are subsequently secreted as NE-derived exosomes enriched with catalytically active cPLA_2_α and the LTB_4_-synthesis machinery.

### Nuclear squeezing promotes NE-MVB formation and pMLCII polarization in primary neutrophils

**Fig. 8.**
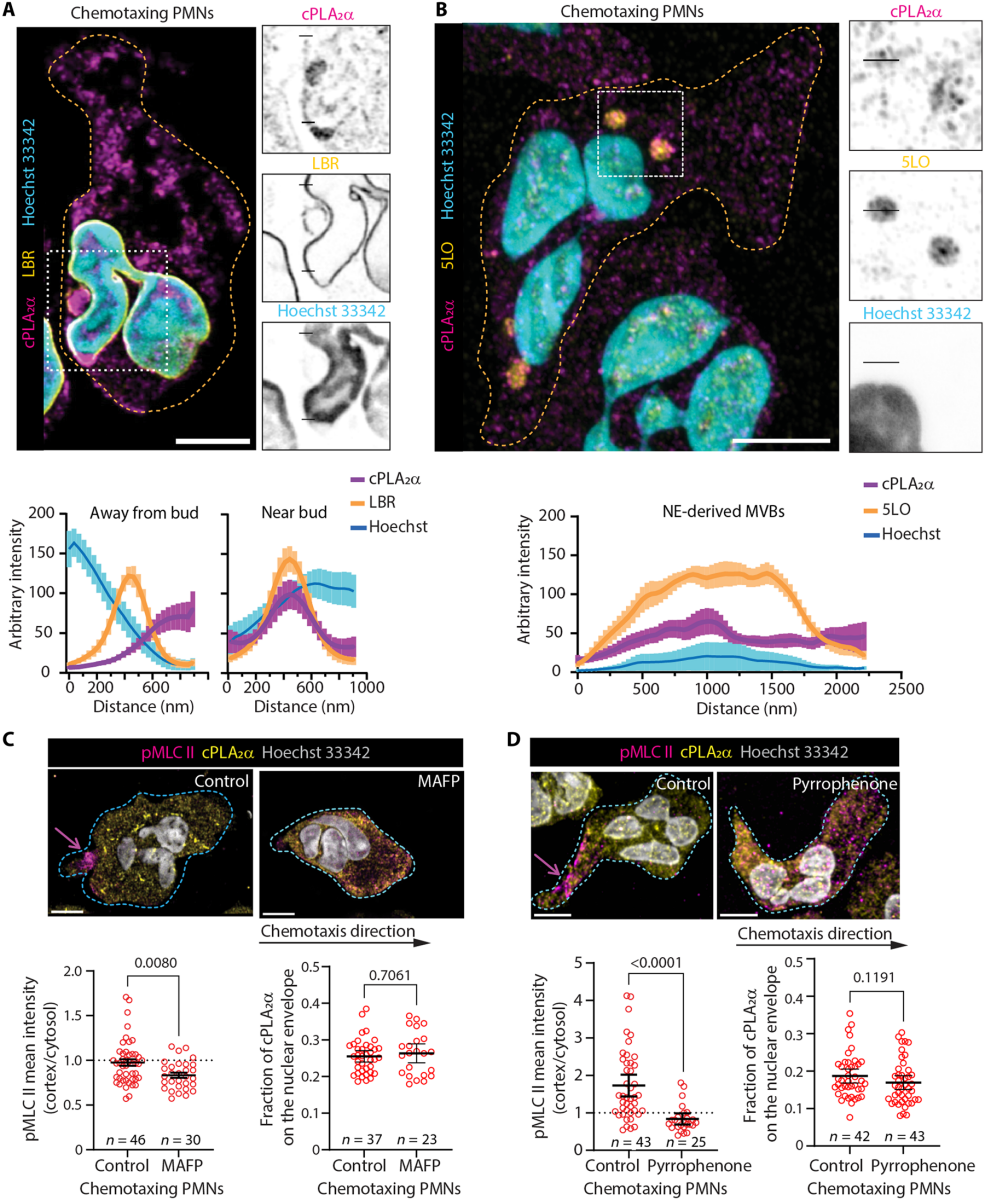
cPLA_2_α activity is required for pMLCII polarization during neutrophil chemotaxis. (**A** and **B**) Representative airyscan microscopy images of PMNs chemotaxing under agarose, immunostained for cPLA_2_α (magenta) and (A) LBR (yellow) or (B) 5-LO (yellow), and costained with Hoechst 33342 (cyan). Orange outlines indicate cell boundaries. The region within the dashed white rectangle is shown on the right as individual inverted grayscale channels. Fluorescence intensity profiles across the lines masked on zoomed insets (A) near NE bud (bottom line) and away from bud (top line) and (B) on the NE-derived cytosolic vesicle are plotted below the zoomed panels. The line profiles are presented as mean ± SEM of 11 NE-budding sites of 9 cells (A), and 10 MVBs from 7 cells (B). *N* = 3. Scale bar, 5 μm. (**C** and **D**) Representative confocal microscopy images (top) of PMNs treated with either DMSO or MAFP (C) and pyrrophenone (D), chemotaxing under agarose, immunostained for pMLC II (magenta), cPLA_2_α (yellow), and costained with Hoechst 33342 (gray). Blue outlines indicate cell boundaries, and the magenta arrow points to polarized pMLC II. Scale bar, 5 μm. Quantification from multiple images is presented as the scatter plot (bottom) showing cortex to cytosol pMLC II intensity and fraction of cPLA_2_α on the NE. Multiple data points (*n*) pooled from two experiments are plotted as a means ± 95% CI, and the *P* values determined using the Mann-Whitney test are presented.

Neutrophils chemotaxing under agarose generate NE-derived MVBs that serve as platforms for LTB_4_ synthesis and chemotaxis signal relay (*16*, *21*). In chemotaxing neutrophils, we observe enrichment of cPLA_2_α at NE-budding sites, marked by LBR positivity and exclusion of Hoechst 33342 staining (Fig. 8A). In contrast, cPLA_2_α is not observed in regions of NE distant from budding sites (Fig. 8A), as confirmed by line profile analysis. In addition, cPLA_2_α is detected on 5LO-positive cytosolic vesicles that likely originate from NE-buds, as shown by line-profile analysis (Fig. 8B).

To test whether cPLA_2_α catalytic activity affects actomyosin contractility, we assessed pMLC II levels in chemotaxing human PMNs treated with the cPLA_2_α inhibitors MAFP (*50*) or pyrrophenone (*51*), or with DMSO as a control. Although NE distribution of cPLA_2_α remains unchanged, MAFP treatment significantly reduces cortical pMLC II levels, particularly at the cell rear (Fig. 8, C and D), indicating that cPLA_2_α-dependent AA production—in addition to its subcellular location—is important for proper actomyosin regulation during chemotaxis.

**Fig. 9.**
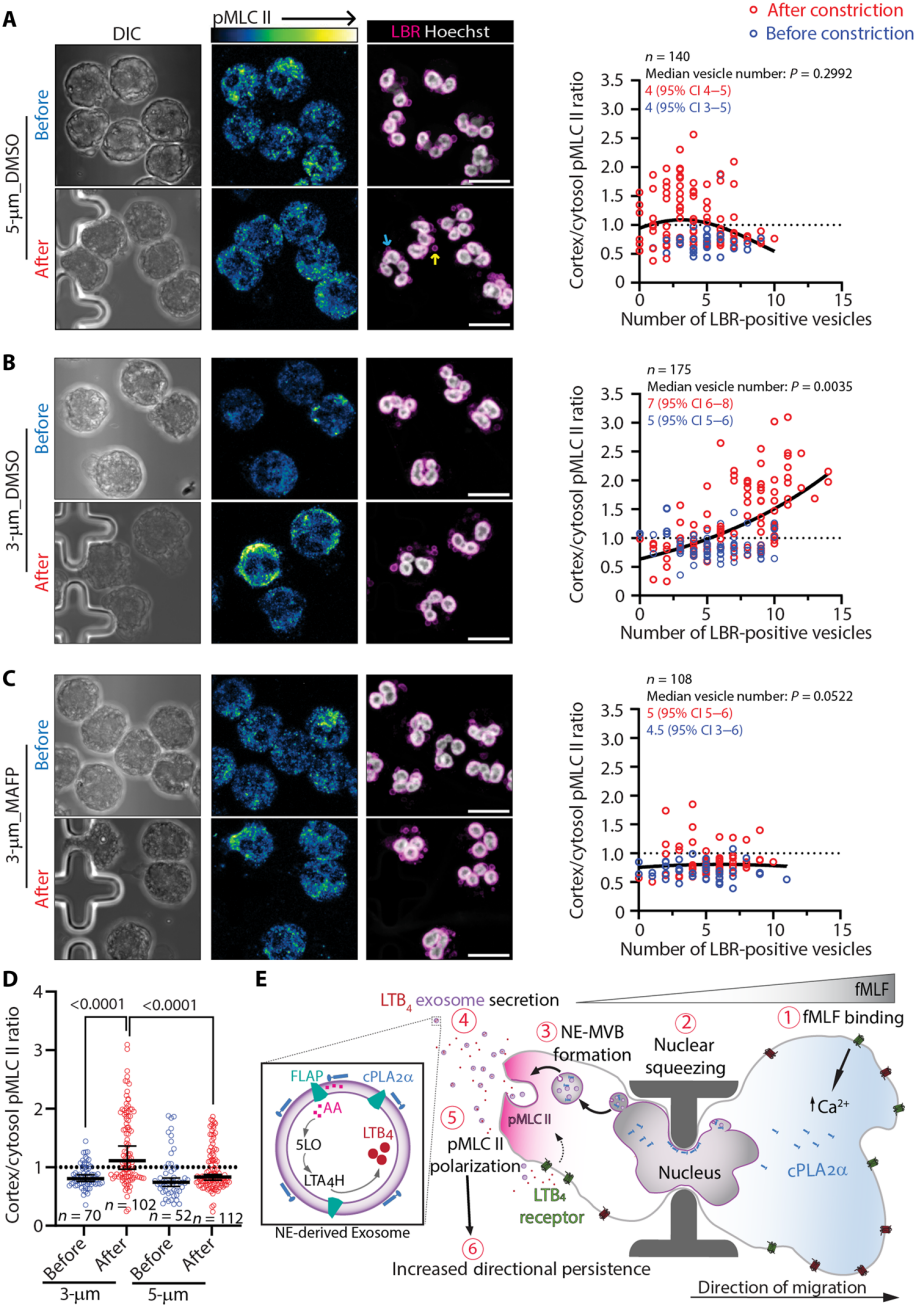
Nuclear squeezing triggers nuclear budding and polarized pMLC II in PMNs. (**A** to **C**) Representative confocal images showing differential interference contrast (DIC), pMLC II (heatmap), and LBR (magenta) with Hoechst (gray) in neutrophils chemotaxing through 5- and 3-μm constrictions under the indicated conditions. Yellow and blue arrows denote NE-bud and NE-derived vesicles, respectively. Scatter plots show cortex/cytosol pMLC II ratios versus number of LBR-positive vesicles per cell (*n* = number of cells from two independent experiments). The dashed line depicts no change in pMLC II ratio, and the solid black line indicates the slope of the nonlinear regression. The median ± 95% CI of the number of LBR-positive vesicles in each condition for before (blue) and after (red) constrictions is mentioned on the graph, with *P* value as calculated using uncorrected Dunn’s test. (**D**) Scatter dot plot of cortex/cytosol pMLC II ratio of Scr dHL60 before and after migration through 3- and 5-μm constrictions, presented as means ± 95% CI, with individual data points (circles, *n*) pooled from two independent experiments. The *P* values are calculated using ordinary one-way ANOVA. (**E**) Schematic illustrating the proposed model whereby nuclear squeezing promotes cPLA_2_α recruitment and packaging onto the exofacial surface of NE-derived exosomes, leading to polarized pMLCII activation and persistent neutrophil chemotaxis.

To directly interrogate the relationship between NE-budding and actomyosin contractility in response to nuclear squeezing, we assessed pMLC II polarity and the presence of NE buds/NE-MVBs in human PMNs chemotaxing within C^3^ devices. In DMSO or MAFP-treated cells, we observe a median of four LBR-positive NE-buds (Fig. 9A, blue arrow) and NE-MVBs per cell (Fig. 9A, yellow arrow) before constriction (Fig. 9, A to C). While a modest increase in cortical pMLC II is observed in a subset of PMNs squeezing through 5-μm constrictions, PMNs squeezing through 3-μm constrictions display a robust spike in cortical pMLC II (Fig. 9, A, B, and D). Notably, this increase in cortical pMLC II strongly coincided with a marked rise in NE-derived vesicle abundance, with cells exhibiting a median of seven NE-derived vesicles following 3-μm constriction (Fig. 9B). In contrast, cPLA_2_α-inhibited PMNs (MAFP-treated) still generate NE-derived buds and vesicles post–nuclear squeeze, confirming that NE budding itself does not require cPLA_2_α catalytic activity. However, these cells failed to exhibit the 3 μm–induced increase in cortical pMLC II (Fig. 9C). Thus, nuclear deformation can trigger nuclear budding, but cPLA_2_α activity is required to translate these mechanical cues to actomyosin polarization. This observation aligns with our findings in HL60-derived neutrophils, where cPLA_2_α-dependent calcium spikes and downstream myosin activation are necessary for maintaining persistent postconstriction chemotaxis (Figs. 3, 5, and 6).

Together, findings from this study support a model in which transient nuclear squeezing during migration through tight constrictions promotes inward curvature of the INM, favored for cPLA_2_α recruitment. Nuclear squeezing, in turn, drives the formation of NE-derived MVBs and release of NE-derived exosomes, which contain catalytically active cPLA_2_α on the outer surface. These cPLA_2_α-enriched exosomes localize LTB_4_ release, which in turn induces GPCR-dependent calcium spikes and MLC II phosphorylation at the cell cortex. The resulting reinforcement of cell polarity and actomyosin contractility enables neutrophils to maintain directional persistence while navigating mechanically restrictive environments (Fig. 9E).

## DISCUSSION

The LTB_4_ signaling pathway plays a central role in neutrophil extravasation (*25*) and directional migration during inflammation (*6*, *23*). Central to this pathway is cPLA_2_α, which catalyzes the release of AA, the precursor for LTB_4_ biosynthesis (*52*). While the importance of cPLA_2_α in neutrophil chemotaxis has been shown, the spatial and mechanical contexts in which its activity becomes critical remain poorly understood. In this study, we reveal that cPLA_2_α not only governs LTB_4_ production but also orchestrates a nuclear mechanotransduction program that enables neutrophils to navigate physical barriers during chemotaxis. Using a custom-engineered cell migration chamber, we show that nuclear squeezing through tight constrictions induces NE remodeling, resulting in cPLA_2_α redistribution, which promotes LTB_4_ synthesis on secreted exosomes. These exosomes, in turn, reinforce directional migration by activating LTB_4_ signaling and actomyosin contractility. Our findings establish a direct mechanistic link between nuclear deformation, lipid signaling, and chemotactic persistence, highlighting cPLA_2_α as a key integrator of mechanical and chemical cues during immune cell navigation.

Although the role of cPLA_2_α in sensing nuclear curvature changes under hypotonic stress or mechanical compression is well established (*8*, *11*, *53*), its influence on steady-state nuclear architecture remained unexplored. We found that dHL60 neutrophils lacking cPLA_2_α, despite undergoing normal differentiation, exhibit reduced nuclear curvature and surface complexity. This altered nuclear morphology is accompanied by increased LMNA/C expression. These observations are consistent with previous reports implicating cPLA_2_α in the regulation of immune-related gene expression in mechanically stimulated mouse dendritic cells (*9*). We also found that cPLA_2_α levels are elevated in *LMNA/C* KO dHL60 cells, establishing that there is a reciprocal up-regulation of LMNA/C upon cPLA_2_α loss. Furthermore, our findings show that cPLA_2_α-deficient neutrophils with high LMNA/C levels exhibit reduced nuclear mechanotransduction, suggesting that cPLA_2_α regulates mechanotransduction, at least in part, by modulating LMNA/C expression in mature neutrophils. Consistent with the role of LMNA in promoting nuclear stiffness (*54*), we found that *cPLA_2_*α KO neutrophils show impaired global nuclear shape remodeling during migration on sADF matrices. The misalignment of nuclei with the underlying substrate topology observed in *cPLA_2_*α KO neutrophils coincides with a decoupling of cellular and nuclear elongation—indicating impaired nuclear mechanosensitivity. Since we found that nuclear volumes are consistent across all migrating cell types on sADF, we reason that increased LMNA expression and nuclear stiffness, rather than changes in volume and NE unfolding (*10*, *11*, *34*), are responsible for the alignment defects observed in polarized *cPLA_2_*α KO neutrophils.

In contrast to aligned collagen fibers of lymph nodes and tumor microenvironments, which are closely mimicked by the organized architecture of sADF (*33*, *55*), the extracellular matrix in inflamed tissue typically consists of disordered collagen networks with variable pore sizes ranging from 0.5 to 10 μm (*56*). This structural heterogeneity makes transient nuclear deformation a frequent occurrence for neutrophils traversing tissues. To model these physiological conditions, we developed a microfluidic device (C^3^) that maintains stable linear chemoattractant gradients while introducing a single, transient constriction during neutrophil chemotaxis. Unlike existing microchannel systems that focus on either mechanotransduction (unidirectional, no choice) or chemotaxis alone, our device enables two-dimensional (bidirectional choice) migration, enabling simultaneous assessment of how transient nuclear squeezing affects directionality, speed, and polarization. Using the C^3^ device, we observed no significant differences in migration distance, speed, or directionality among Scr, *cPLA_2_*α KO, and GFP-cPLA_2_α neutrophils migrating through 5-μm constrictions. Given that the average nuclear diameter of dHL60 neutrophils is ~4 μm, 5-μm constrictions do not impose substantial nuclear deformation and thus recapitulate behaviors observed in cells chemotaxing under agarose. Notably, all three cell types showed comparable efficiency in crossing 3-μm constrictions, indicating that neither cPLA_2_α deficiency nor elevated LMNA/C levels impair neutrophil transit through narrow spaces. This contrasts with the sADF setup, where large-scale nuclear remodeling is required to align the nucleus with the substrate. Together, these findings suggest that transient, small-scale nuclear squeezing in the C^3^ device is governed by local NE curvature changes rather than global nuclear shape remodeling.

After emerging from 3-μm constrictions, *cPLA_2_*α KO neutrophils exhibit significantly reduced migration distance, speed, and directionality compared to Scr and GFP-cPLA_2_α expressing neutrophils, underscoring a critical role for cPLA_2_α in postconstriction migratory performance. *LMNA/C* KO cells, which express elevated levels of cPLA_2_α, display higher postconstriction persistence than Scr controls in a manner that is dependent on cPLA_2_α activity. Notably, reexpression of GFP-cPLA_2_α in *cPLA_2_*α KO neutrophils restores this behavior, but the rescue is reversed when LTB_4_ synthesis is pharmacologically inhibited, highlighting that the functional contribution of cPLA_2_α depends on its catalytic role in LTB_4_ production. We previously demonstrated that LTB_4_ signaling mediates MLC II phosphorylation and drives cell polarization and directional migration toward fMLF (*24*). Here, we show that calcium spikes as well as phosphorylation of MLC II at the cell cortex occur upon squeezing through 3-μm constrictions and are absent in *cPLA_2_*α KO neutrophils, leading to impaired cell polarity and compromised chemotactic persistence. This is consistent with earlier biochemical studies showing that full activation of phospholipase A_2_ in human neutrophils requires both calcium influx and LTB_4_ production, suggesting a positive feedback loop in which LTB_4_-dependent cPLA_2_α activity couples eicosanoid synthesis to sustain calcium signaling (*57*). Our findings support a model in which nuclear squeezing synchronizes calcium spikes and facilitates cPLA_2_α enrichment at curvature-sensitive regions of the INM, enabling its incorporation into NE-MVBs and subsequently into LTB_4_-producing exosomes. The released LTB_4_ then binds to LTB_4_ GPCRs and acts in an autocrine and/or paracrine manner to promote MLC II phosphorylation and sustain directional migration. Given the pivotal role of LTB_4_ in amplifying chemotactic signaling, this mechanism offers insight into the rapid, directed migration of neutrophils in vivo, where cells must traverse dense, collagen-rich tissues to reach sites of saturating chemoattractant concentrations (*6*). We also observed a notable morphological transition, from a pseudopod-driven amoeboid mode to a fan-shaped, keratocyte-like migration pattern, exclusively when GFP-cPLA_2_α neutrophils passed through 3-μm constrictions. In *Dictyostelium discoideum*, both amoeboid and keratocyte-like migration modes coexist and are dynamically regulated by external cues (*39*, *58*). Cells in the keratocyte-like state typically adopt a broad, laterally expanded lamellipodium with strong frontal actin protrusions and stable, rear-localized actomyosin (Myo II) contractility (*38*, *59*, *60*). This migration mode is supported by moderate substrate adhesion, similar to what is observed during neutrophil infiltration in vivo (*6*, *61*), whereas lower or higher adhesion levels tend to favor amoeboid or slower mesenchymal migration modes, respectively (*5*, *62*).

Ceramide and its metabolite ceramide-1-phosphate enhance cPLA_2_α membrane recruitment and catalytic activity by interacting with its calcium-dependent C2 domain (*63*, *64*).This provides a mechanistic basis for cPLA_2_α enrichment at ceramide-rich domains within the INM, where nSMase1 localizes and NE budding is initiated (*16*). Moreover, cPLA_2_α-dependent AA release has been proposed to activate nSMase in vitro (*65*), suggesting a reciprocal lipid-regulatory loop between these enzymes. However, we found that while cPLA_2_α is present in NE-derived exosomes, cells lacking cPLA_2_α produce the same number of 5LO/FLAP-positive exosomes under nonconstrictive conditions. Similarly, while PMNs migrating through 3-μm constrictions display more NE-MVBs and polarized pMLCII after squeezing, cPLA_2_ inhibition prevents the constriction-induced enhancement, while NE-MVB levels remain comparable to preconstriction. Together, these findings indicate that while cPLA_2_α activity is not required for NE budding per se, under mechanical confinement, its activation converts nuclear deformation into a lipid-signaling response that reinforces polarity. The dependence of cPLA_2_α on calcium and LTB_4_ for full activation (*57*), the ability of AA to enhance nSMase activity (*65*), and the suppression of constriction-induced localized pMLC II upon cPLA_2_α inhibition collectively suggest that active cPLA_2_α promotes the lipid-order transitions that enhance NE-budding and exosomal LTB_4_ release under constrictive environments. Thus, cPLA_2_α functions as a mechanosensitive lipid enzyme that amplifies confinement-induced signaling by coupling membrane curvature with leukotriene synthesis to sustain directional migration.

Trypsin sensitivity of exosome-associated cPLA_2_α indicates that its exposure on the exofacial surface aligns with its INM localization in migrating neutrophils. We propose that upon chemoattractant stimulation, nuclear cPLA_2_α, capable of sensing high positive curvature, is selectively recruited to nascent intraluminal vesicles budding from the INM (Fig. 9E). These cPLA_2_α-containing exosomes then serve as a local hub for rapid LTB_4_ production and secretion. This model is supported by previous findings linking nuclear cPLA_2_α enrichment with AA release and LTB_4_ production in okadaic acid–treated macrophages (*66*) and phosphatase-treated cancer cells (*67*), highlighting a conserved role for nuclear cPLA_2_α in curvature sensing and eicosanoid signaling. While it remains to be determined whether the cytosolic pool of cPLA_2_α translocates to the nuclei of activated neutrophils, the cytoplasmic pool has been implicated in prostaglandin synthesis—a distinct eicosanoid class—on lipid droplets derived from the tubular ER (*68*).

Together, our data suggest a previously unknown function for nuclear cPLA_2_α in establishing neutrophil polarity and promoting a transition toward keratocyte-like migration. This switch appears to be tightly linked to localized LTB_4_ production and downstream phosphorylation of MLC II, a key effector of MyoII activity. Given that rear MyoII contractility is essential for maintaining front-rear polarity and directional persistence, we propose that NE remodeling serves as a mechanochemical relay that links nuclear deformation to the self-amplifying cPLA_2_α-LTB_4_-pMLC II axis that sustains long-range chemotactic coordination. These findings highlight a previously unrecognized mechanism by which confined migration triggers an exosome-mediated signaling cascade to reinforce migratory behavior. Future studies will help dissect how this axis governs migratory mode transitions in immune cells across different tissue contexts.

## MATERIALS AND METHODS

### Ethics statement

Human neutrophils were isolated from peripheral blood obtained by venipuncture of anonymous healthy donors recruited by the Platelet, Pharmacology, and Physiology Core facility at the University of Michigan. The blood collection procedure adhered to the institutional review board protocol (IRB #HUM00107120), approved explicitly for supplying deidentified blood for research. Consequently, the authors do not have access to Health Insurance Portability and Accountability Act (HIPAA) information. All participants consented to provide their blood for research purposes and were financially compensated. All the deidentified healthy blood donors verified no intake of nonsteroidal anti-inflammatory drugs for 48 hours before blood collection.

### Cell culture

The human myeloid leukemia-derived promyelocytic cell line HL60 was obtained from American Type Culture Collection (ATCC, CCL-240) and maintained in RPMI 1640 (Gibco, 11875-093) medium containing 10% heat-inactivated fetal bovine serum (FBS), 20 mM Hepes (pH 7.2), and penicillin-streptomycin antibiotic cocktail (100 U/ml; Thermo Fisher Scientific, #15-140-122). To generate neutrophil-like cells, HL60 cells were differentiated in culture medium containing 1.3% DMSO for 6 days with a change to fresh medium every other day as described by Saunders *et al.* (*29*)

Human embryonic kidney (HEK) 293T cells obtained from ATCC (CRL-3216), cultured in Dulbecco’s modified Eagle’s medium supplemented with 10% FBS, were used to generate lentiviral particles for gene KO expression in HL60 cell lines. Lentiviral packaging plasmids pVSVG and psPax2, along with pLentiCRISPR V2 vector expressing either Scr single guide RNA (sgRNA), cPLA_2_α sgRNA, or pCDH MSCV MCS EF1 neomycin vector expressing cPLA_2_α fused with enhanced GFP (eGFP) in-frame at its N-terminal, were transfected to HEK293T at a ratio of 1:2:4 using Lipofectamine 3000 transfection reagent. The culture supernatant containing lentiviral particles was collected after 48 and 72 hours posttransfection and pooled. Lentiviral particles were concentrated by incubating supernatants in 1× lentivirus concentrator [4× stock, 40% w/v polyethylene glycol, molecular weight 8000 (PEG-8000), and 1.2 M NaCl in 1× phosphate-buffered saline (PBS)] overnight at 4°C and precipitated at 1500*g* for 45 min at 4°C. The concentrated virus was resuspended in RPMI 1640 containing hexadimethrine bromide (polybrene) (8 μg/ml; Sigma-Aldrich, H9268-5G) and added to HL60 cells. The clones expressing the construct were selected in puromycin (pLentiCRISPR V2) (2 μg/ml) or G418 (pCDH MSCV MCS EF1) (1 mg/ml), dilution plated for single-cell cloning, and verified using Western blotting and genetic sequencing.

### Plasmid constructs

The cPLA_2_α sgRNA, ACACCACTACCGTAAACTTG, was cloned into the pLentiCRISPR V2 plasmid, which was a gift from the Zhang laboratory. pCDH-puro-GFP-cPLA_2_α construct was cloned using the NEB Gibson assembly kit (NEB E5510). cPLA_2_α was amplified from GenScript plasmid pCDNA3.1-cPLA_2_α (Clone ID Ohu19957) using 5′-ctgtacaagATGTCATTTATAGATCCTTACCAG-3′ and 5′-ccctcagcggccgcggatccTGCTTTGGGTTTACTTAGAAAC-3′, and GFP was amplified from FPR1-eGFP plasmid from Subramanian *et al.* (*24*) using 5′-gagctagagctagcgaattcGCCACCATGGTGAGCAAG-3′ and 5′-taaatgacatCTTGTACAGCTCGTCCATGC-3′ primers. GFP- cPLA_2_α was amplified from pCDH-puro-GFP-cPLA_2_α construct using 5′-gcgggcGCTAGCATGGTGAGCAAGGGCGAGG-3′ and 5′-gcgcggcGCGGCCGCctaTGCTTTGGGTTTACTTAG-3′ primers and cloned into the NheI and NotI sites in pCDH MSCV MCS EF1 neomycin vector. All cloned constructs were confirmed by Sanger sequencing.

### Isolation of human neutrophils

Blood was donated by healthy males and females who had not taken aspirin for 7 days and nonsteroidal anti-inflammatory drugs (NSAIDs) for 48 hours. Blood was collected by venipuncture from the Platelet Pharmacology and Physiology Core at the University of Michigan. Neutrophils were purified using dextran-based sedimentation followed by histopaque density gradient centrifugation as described earlier (*69*). Briefly, whole blood was incubated with an equal volume of 3% dextran (Sigma-Aldrich, D1037) in 0.9% NaCl for 1 hour at 37°C to sediment erythrocytes. One volume of Histopaque-1077 (Sigma-Aldrich, 10771) was underlaid to three volumes of plasma containing monocytes, lymphocytes, and neutrophils and centrifuged at 400*g* for 20 min without break to separate neutrophils from peripheral blood mononuclear cells. Residual RBCs were lysed using ACK lysis buffer (Gibco, A10492-01, 100 ml). Isolated neutrophils are resuspended in mHBSS [150 mM NaCl, 4 mM KCl, 1.2 mM MgCl_2_, 5 mM glucose, and 20 mM Hepes (pH 7.2)] This protocol yields >95% neutrophils.

### Under agarose chemotaxis assay and chemotaxis analysis

Chemotaxis assay was performed as previously described (*29*). Briefly, 0.5% SeaKem ME agarose (Lonza, 50010) in 1:1 Dulbecco’s Phosphate-Buffered Saline (DPBS) (Gibco, 14190-144) and 1× mHBSS was solidified in either 35-mm glass bottom (Celvis) or eight-well chamber glass bottom chambers (Celvis C8-1.5H-N) precoated with 1% bovine serum albumin (BSA, 50 ml; Sigma-Aldrich, A7979) in DPBS. Two wells of 1-mm diameter each were carved 2 mm apart. Differentiated HL60 cells were resuspended in washed in DPBS, counted, resuspended at a density of 10 million/ml, and stained using Hoechst 33342 nuclear stain (1 μg/ml, Invitrogen, H21492) for 15 min at 37°C at 10 RPM. fMLF (100 nM) diluted in 1× mHBSS was added to one well, and 50,000 stained cells in 5 μl of mHBSS were added to another well. Time-lapse images were acquired at 30-s intervals for 1.5 hours using a 10× objective of a fluorescent microscope equipped with an environment-controlled unit set at 37°C.

Using the TrackMate plugin, all the migrating cells (200 to 1000) were tracked on the Hoechst 33342 channel. Spot statistics were downloaded from TrackMate analysis and uploaded into the chemotaxis analysis code in MATLAB R2021a. Tracks with less than 100-μm final distance on the *x* axis were excluded from the final analysis. The average of chemotaxis parameters for each experiment was plotted using GraphPad Prism.

### Expansion microscopy and immunofluorescence staining

dHL60 cells or PMNs were allowed to migrate under agarose toward 100 nM fMLF for 1 hour and fixed with 4% paraformaldehyde (PFA) (Electron Microscopy Sciences, 15174) diluted in 1× mHBSS at 37°C for 20 min. The agarose was scooped out, and the cells were fixed and blocked in staining solution (0.5% saponin and 2% goat serum in PBS) for 1 hour at room temperature. Cells were stained overnight at 4°C using primary antibodies diluted in a staining solution. The antibodies and their dilutions used for normal immunofluorescence staining are rabbit anti-GFP (1:2000, Thermo Fisher Scientific, A6455), rabbit anti-LBR (1:500, Abcam, 32535), rabbit anti-FLAP (1 μg/ml, Abcam, 85227), mouse anti-cPLA_2_α (1:100, Santa Cruz Biotechnology, 376618), rabbit anti-5LO (1:200: Abcam, 169755), and rabbit anti-phospho MLC II (1:100, Cell Signaling Technology, 3674S). Stained cells were washed once with 0.2% saponin in DPBS, followed by DPBS twice for 5 min each, and by incubation with Alexa Fluor–tagged secondary antibodies (1:500) in staining solution for 1 hour at room temperature. The cells were washed with DPBS thrice, overlaid with 150 μl of ImmuMount mounting media (Thermo Fisher Scientific, 9990402), and stored at 4°C until imaging on a Zeiss 880 confocal/airyscan microscope.

For 4× expansion microscopy, GFP-cPLA_2_α dHL60 cells were allowed to migrate for 1 hour at 37°C under agarose on a BSA-coated 22 mm–by–22 mm coverslip (#1.5) placed in a 35-mm dish. The cells were fixed with 1 ml of 4% PFA and 0.05% glutaraldehyde in PHEM buffer [60 mM Pipes, 25 mM Hepes, 10 mM EGTA, and 2 mM MgCl_2_ (pH 6.9)] at 37°C for 20 min. After gently removing the agarose, the coverslip-containing dish was incubated with 2 ml of anchoring solution (2% acrylamide and 1.4% formaldehyde in PBS) for 3 hours at 37°C. For gelation, the coverslip was removed from the gelation chamber, placed in a 35-mm dish, and incubated with 2 ml of denaturation buffer [200 mM SDS, 200 mM NaCl, and 50 mM tris base (pH 6.8)] with shaking at room temperature until the gel detached, followed by denaturation at 85°C for 90 min. The gel was expanded in double-distilled water (10 ml, five times) by incubating it at room temperature with shaking in a 10-cm dish for 20 min each time. The gel was then incubated in PBS containing Hoechst 33342 to visualize cells. Gel pieces (~1 cm^2^) containing migrated cells were excised and transferred to 1.5-ml tubes containing 250 μl of antibody staining solution (PBS with 2% BSA) and the following primary antibodies: mouse anti-calnexin (1:50, Proteintech, 66903-1-Ig), mouse anti-cPLA_2_α (1:50, Santa Cruz Biotechnology, 376618), rabbit anti-GFP (1:100, Abcam, 290), and rabbit anti-LBR (1:50, Abcam, 32535). Incubation was performed overnight at 4°C with end-over-end rotation at 10 RPM. Gels were washed with 500 μl PBS containing 0.1% Tween-20 three times for 10 min each with rotation. Secondary antibody incubation was carried out for 6 hours at room temperature, followed by three washes with PBS + 0.1% Tween-20 and a final PBS wash. The gel was reexpanded in double-distilled water three times at room temperature for 20 min each, followed by overnight expansion at 4°C to reach saturation. The expanded gel was immobilized (cell side down) on a poly-l-lysine–coated 12-mm glass-bottom 35-mm dish. Gels were gently pressed with a soft brush to prevent drift, surrounded with 100 μl of double-distilled water to prevent shrinkage, and overlaid with a 22 mm–by–22 mm coverslip to stabilize the sample. Imaging was performed using a Zeiss LSM 880 with Airyscan and a 63× (1.4 numerical aperture) oil objective. The effective resolution postexpansion was ~30 to 40 nm laterally and ~100 nm on the *z* axis.

### Intact nuclei isolation and immunostaining

This protocol was used as described previously in Arya *et al.* (*16*)*.* Briefly, 50 million dHL60 were resuspended in 1 ml 1× mHBSS. To inhibit proteases, cells were pretreated with 2 mM AEBSF hydrochloride (Pefabloc, Thermo Fisher Scientific, AC328110010) for 15 min at 37°C, before stimulation with 100 nM fMLF for either 15 or 30 min at 37°C while rotating at 10 RPM. Cells were pelleted at 6000*g* for 30 s, and the proteins were cross-linked using 10 mM dimethyl pimelimidate (DMP, TCL Chemicals, D4476) in 1× mHBSS for 15 min at 37°C, followed by quenching free DMP using 20 mM tris-Cl (pH 8.0) for 10 min at room temperature. The plasma membrane was partially lysed twice to separate nuclei and cytosol by triturating cells (50 million/ml) 10 times in ice-cold hypotonic lysis buffer [10 mM Hepes (pH 7.2), 4 mM MgCl_2_, 25 mM NaCl, 1 mM dithiothreitol (DTT), and 0.1% NP-40], using 1-ml pipette at a density of 50 million cells/ml followed by centrifugation until speed reaches 16,000*g*. Supernatants from the first lysis were collected as the cytosolic fraction. The pellet after the second centrifugation was washed twice with ice-cold Barnes solution [85 mM KCl, 85 mM NaCl, 2.5 mM MgCl_2_, and 5 mM trichloroacetic acid (TCA) (pH 7.2)] to remove residual ER fragments (microsomes) from the nuclei. Supernatants from the first wash were collected as the Barnes (ER) fraction. For the Western blot analysis, all fractions were resuspended in 1× XT sample buffer, boiled at 95°C for 10 min, and loaded in equal volume. For immunofluorescent staining, purified nuclei corresponding to that of 1 million cells were resuspended in 1 ml of ice-cold nuclei resuspension buffer [10 mM Hepes (pH 7.2), 4 mM MgCl_2_, 150 mM NaCl, 1 mM DTT, and 250 mM sucrose] and added to poly-l-lysine (Sigma-Aldrich, P4832)–coated #1.5 glass coverslips and centrifuged at 500*g* for 5 min at 4°C. The isolated nuclei were then fixed using 4% PFA in resuspension buffer for 10 min at room temperature, followed by primary antibody staining overnight at 4°C in DPBS containing 2% goat serum. The antibodies used are mouse anti-ceramide (1:200, Sigma-Aldrich, C8104-50TST), rabbit anti-GFP (1:500, Thermo Fisher Scientific, A6455), and rabbit anti-FLAP (1:200, Novus NB300-891).

### Nuclear DRM extraction

For lipid-ordered microdomains extraction, membranes of ~50 million isolated nuclei were solubilized in 650 μl of ice-cold TNE buffer [50 mM tris-Cl (pH 7.4), 150 mM NaCl, and 5 mM EGTA] containing 4 mM MgCl_2_ and 1% Triton X-100. The suspension was homogenized by passing through a 23G needle 30 times, and the homogenate was incubated on ice for 30 min. Supernatants containing solubilized nuclear membranes and nucleoplasm were collected at 110*g* for 10 min at 4°C, and the pellet (chromatin/nucleoskeleton) was discarded. Supernatants were adjusted to 40% optiprep using 60% OptiPrep stock solution (Sigma-Aldrich, D1556) to a final volume of 2 ml and overlayed with 7 ml of 30% iodixanol, 2 ml of 20% iodixanol, and followed by 1 ml of 5% iodixanol solution in TNE buffer, in 13-ml ultracentrifuge tubes (Beckman, 331372). After centrifugation at 150,000*g* for 16 hours at 4°C, 16 750-μl fractions from the top were collected. The proteins in collected fraction were precipitated using TCA-acetone method, air dried, and were resuspended in 80 μl of 1× XT sample buffer (Bio-Rad, 1610791) under reducing conditions, boiled at 95°C for 10 min and loaded on Criterion XT 4 to 12% bis-tris gel (Bio-Rad, 3450124) for electrophoresis. The electrophoresed proteins were transferred to 0.2-μm polyvinylidene difluoride membrane, blocked using 1× fish gelatin (Thermo Fisher Scientific, NC0382999) in tris-buffered saline containing 0.1% Tween-20 (Thermo Fisher Scientific, 337-500), and probed for specific proteins using antibody against FLAP (1 μg/ml, Abcam, 85227), flotillin 2 (1:1,000, CST, 3436), nSMase1 (1:500, CST, 3867), and cPLA_2_α (1:1,000, Santa Cruz Biotechnology, sc-376618), using electrochemiluminescence capture on photographic films.

### Generation of sADF fibslips

DexVS fiber mats were synthesized using electrospin technology as described previously by Loesel *et al.* (*33*). Briefly, DexVS (0.6 mg/ml) dissolved in dimethylformamide: MilliQ water (1:1) was mixed with lithium phenyl-2,4,6-trimethylbenzoylphosphinate (LAP, 100 mg/ml), 0.75 mM methacryloxyethyl thiocarbamoyl rhodamine B, and 5 vol % glycidyl methacrylate to generate an electrospinning solution. DexVS solution was electrospun in a humidity-controlled glove box at 30 to 35% relative humidity and 0.2 ml/hour flow rate. To create aligned fibers, an 18-mm^2^ glass coverslip (Thermo Fisher Scientific, 12546) was placed between two parallel copper electrodes set to −4.0 kV. The stainless-steel needle containing the polymer solution was situated 7 cm from the collection surface and connected to the voltage source set to +4.0 kV. Electrospun fibers were deposited onto the coverslip for 5 min to achieve the desired thickness of the fiber mat. The fiber mats were primarily crosslinked under ultraviolet light at 100 mW/cm^2^ for 20 s to stabilize the fibers. The fiber-coated coverslips were glued to the bottom of a modified 12-well plate using SYLGARD 164 Silicone Elastomer kit (Dow, 0.4028273). The 12-well plates (Thermo Fisher Scientific, FB012928) were modified by drilling 15-mm^2^ holes at the bottom of a polystyrene 12-well plate using a Dremel 7760 tool.

Before the experiment, the fiber mats were functionalized with 2.5% (w/v) heparin methacrylate dissolved in LAP solution (1 mg/ml) using 100 mW/cm^2^ ultraviolet light exposure for 20 s. The 12-well plates are sterilized using 70% ethanol for 10 min, followed by coating with fibrinogen (10 μg/ml; Sigma-Aldrich, 4129) in DPBS for 1 hour at 37°C, before plating cells.

### C^3^ fabrication and chemotaxis assays

We first generated a computational model of C^3^ using AutoCAD (Autodesk). The master molds were then fabricated using the standard photolithography method using SU-8 photoresist (MicroChem) following the manufacturer’s protocol. Two masks were used to fabricate the multiple heights, one for the migration channels (5 μm high) and the other for the cell/chemoattractant channels (100 μm high). PDMS (Sylgard 184, Dow Corning) was prepared with the 10:1 elastomer-to-curing agent ratio. PDMS was poured on the channel molds and cured at 80°C for 4 hour to polymerize before peeling to generate a PDMS layer of C^3^. The PDMS layer and the substrate (#1.5 glass coverslip 22 mm by 22 mm) were activated by oxygen plasma treatment to facilitate bonding. The devices are stored at room temperature in a moisture-free environment. Before the experiment, the C^3^ was desiccated at 150°C for 5 min and sterilized for 20 min by ultraviolet exposure in a laminar flow hood. C^3^ channels and migration chamber were then filled with 1% BSA in DPBS and incubated for 1 hour at 37°C to coat the glass and PDMS surface and avoid excessive cell adhesion on negatively charged surfaces, which impedes migration. The channels were then washed with DPBS and then filled with 1× mHBSS for the experiment. Ten microliters of dHL60 cell suspension at a density of 20 million cells/ml was added to the cell inlet port, and 10 μl of chemoattractant (100 nM fMLF in mHBSS) was added to chemoattractant inlet port. Three microliters of cell suspension and chemoattractant each was aspirated from their respective outlets. The cells were allowed to chemotax toward increasing chemoattractant concentration in the migration chamber and imaged using a 10× objective on a Zeiss Colibri microscope fitted with an environment control chamber maintained at 37°C and >90% humidity. Image acquisition was automated using MetaMorph software. For downstream amoeboid-keratocyte transition analysis, cells were stained with CellMask orange (1 μM) in addition to Hoechst 33342 (1 μg/ml) used for tracking analysis. For the measurement of calcium fluxes, cells were stained with Fluo4-AM (1 μM) and Hoechst.

### Microscopy and image analysis

Fixed and immunostained cells/isolated nuclei were imaged using the Plan Apochromat 63×/1.4 Oil DIC M27 objective on Zeiss LSM 880, AxioObserver equipped with AiryScan Superresolution mode. For colocalization analysis, regions of interest of isolated nuclei were thresholded using maximum entropy parameters, and Mander’s colocalization coefficients were determined using the Coloc2 analysis plugin in FIJI. The values were plotted using GraphPad Prism software. Outliers were calculated using the GraphPad Prism ROUT test (*Q* = 1%) and were excluded from the final statistical analysis using the Mann-Whitney test.

### Exosome isolation, LTB_4_ ELISA, and trypsin protection assay

Exosome isolation was performed according to the guidelines described by Welsh *et al.* (*70*) . Scr, *cPLA_2_*α KO, and GFP-cPLA_2_α dHL60 neutrophils were stimulated with 100 nM fMLF in 1× mHBSS containing deoxyribonuclease I (10 U/ml, Sigma-Aldrich, DN25) for 30 min at 37°C. The cells were pelleted at 500*g* for 5 min at 4°C, and supernatants were collected and centrifuged again at 4000*g* for 20 min to remove microvesicles and apoptotic bodies. The supernatant was mixed 1:1 with 16% PEG-6000 (Bio Basic PB0432) dissolved in 20 mM Hepes (pH 6.9) and 500 mM NaCl to precipitate extracellular vesicles (EVs) at 4°C for 36 hours, followed by centrifugation at 4000*g* at 4°C for 1 hour. The PEG-precipitated EVs were washed by resuspending the pellet in 5 ml of ice-cold PBS and centrifugation at 100,000*g* for 1 hour at 4°C in the Beckman SW55Ti rotor. The concentrated EVs were resuspended in 1 ml of 250 mM sucrose and 20 mM tris-Cl (pH 7.4), overlayed on top of optiprep gradients, and centrifuged at 100,000*g* for 16 hours at 4°C using a Beckman Sw41Ti rotor. The optiprep gradients prepared in 250 mM sucrose and 20 mM tris-Cl (pH 7.4), were layered starting from the bottom as 3 ml of 40% optiprep, 3 ml of 20% optiprep, 3 ml of 10% optiprep, and 2 ml of 5% optiprep. The fractionated exosomes were collected as 12 fractions of 1 ml each, starting from the top (lower to higher density). Exosome-enriched fractions four to nine (iodixanol density 1.083 to 1.142 g/ml) were pooled and diluted to 13 ml with PBS, followed by centrifugation at 100,000*g* for 1 hour at 4°C. The purified exosomes were used to assess exosomal LTB_4_ content and determine the distribution of exosome-associated proteins.

LTB_4_ enzyme-linked immunosorbent assay (ELISA) kit (Cayman Chemicals, 520111) was used to assess LTB_4_ levels within the isolated exosomes homogenized in 100 μl of ELISA buffer using a 3-mm diameter sonicator probe at an amplitude of 20% with 2-s on/off cycles for a total of 10 cycles on ice. To detect LTB_4_ concentrations within the linear range, 50 μl of concentrated homogenate was diluted 4× in ELISA buffer, and LTB_4_ levels were quantified according to the manufacturer’s instructions. The values obtained were plotted using GraphPad Prism.

To determine the distribution of proteins within the exosomes, the isolated exosomes were resuspended in mHBSS supplemented with 1 mM CaCl_2_, volumetrically divided into two equal fractions, and one fraction treated with trypsin (50 μg/ml; Thermo Fisher Scientific, 25200072) for 30 min at 37°C. The trypsin was inactivated by diluting the exosomes in mHBSS supplemented with AEBSF hydrochloride, followed by centrifugation at 120,000*g* for 1 hour at 4°C to pellet the exosomes. The pelleted exosomes were lysed in 1× XT sample buffer at 95°C for 10 min, and equal volumes were loaded on a Criterion XT 4 to 12% bis-tris electrophoresis gel for Western blotting.

### Generation of whole-cell lysate for Western blotting

dHL60 neutrophils were washed once with DPBS, resuspended in mHBSS, and treated with 2 mM Pefabloc for 15 min while rotating. Cells were pelleted at 6000*g* for 30 s and resuspended in 1× Laemmli sample buffer (Thermo Fisher Scientific, AAJ61337AD) diluted in PBS. Samples were boiled at 95°C for 10 min, and lysate volume equivalent to 250,000 cells was loaded on a 4 to 20% tris-glycine gel (Invitrogen XP04205BOX) for electrophoresis. The proteins were transferred to 0.2-μm nitrocellulose membrane (MDI, SCNX8401XXXX101), blocked using 1× fish gelatin (Thermo Fisher Scientific, NC0382999) in 0.1% Tween-20 containing tris-buffered saline, and probed for specific proteins using antibody against cPLA_2_α (1:1,000, Santa Cruz Biotechnology, sc-376618), FLAP (1 μg ml^−1^, Abcam 85227), 5LO (1:1000, Abcam, ab169755), LTA_4_H (1:1000 Protein Tech, 13662-1-AP), and glyceraldehyde-3-phosphate dehydrogenase (1:1000, Santa Cruz Biotechnology).

### Statistics and reproducibility

Unless otherwise indicated, all data shown are from at least three independent biological replicates. When the analysis focuses on single-cell phenotypes over time, pooled data points (*n*) from two independent experiments (*N*) are presented to assess both intra-cell and population-level variability. Appropriate statistical tests were performed to evaluate significance, as indicated in the corresponding figure legends.
