# Supplementary material for: cPLA2α targeting to exosomes connects nuclear deformation to LTB4-signaling during neutrophil chemotaxis
Source: Sci Adv. 2026 Feb 20;12(8):eaea2784. doi: 10.1126/sciadv.aea2784 (PMC12922757; doi:10.1126/sciadv.aea2784)
Supplement: Supplementary file 1 — Figs. S1 to S3 Legends for movies S1 to S4 [file sciadv.aea2784_sm.pdf]

Science Advances
AAAS

Supplementary Materials for

cPLA$_2$α targeting to exosomes connects nuclear deformation to LTB$_4$-signaling during neutrophil chemotaxis

Subhash B. Arya *et al.*

Corresponding author: Carole A. Parent, parentc@umich.edu

Sci. Adv. **12**, eaea2784 (2026)
DOI: 10.1126/sciadv.aea2784

The PDF file includes:

Figs. S1 to S3
Legends for movies S1 to S4

Other Supplementary Material for this manuscript includes the following:

Movies S1 to S4

1. Supplementary figures and legends

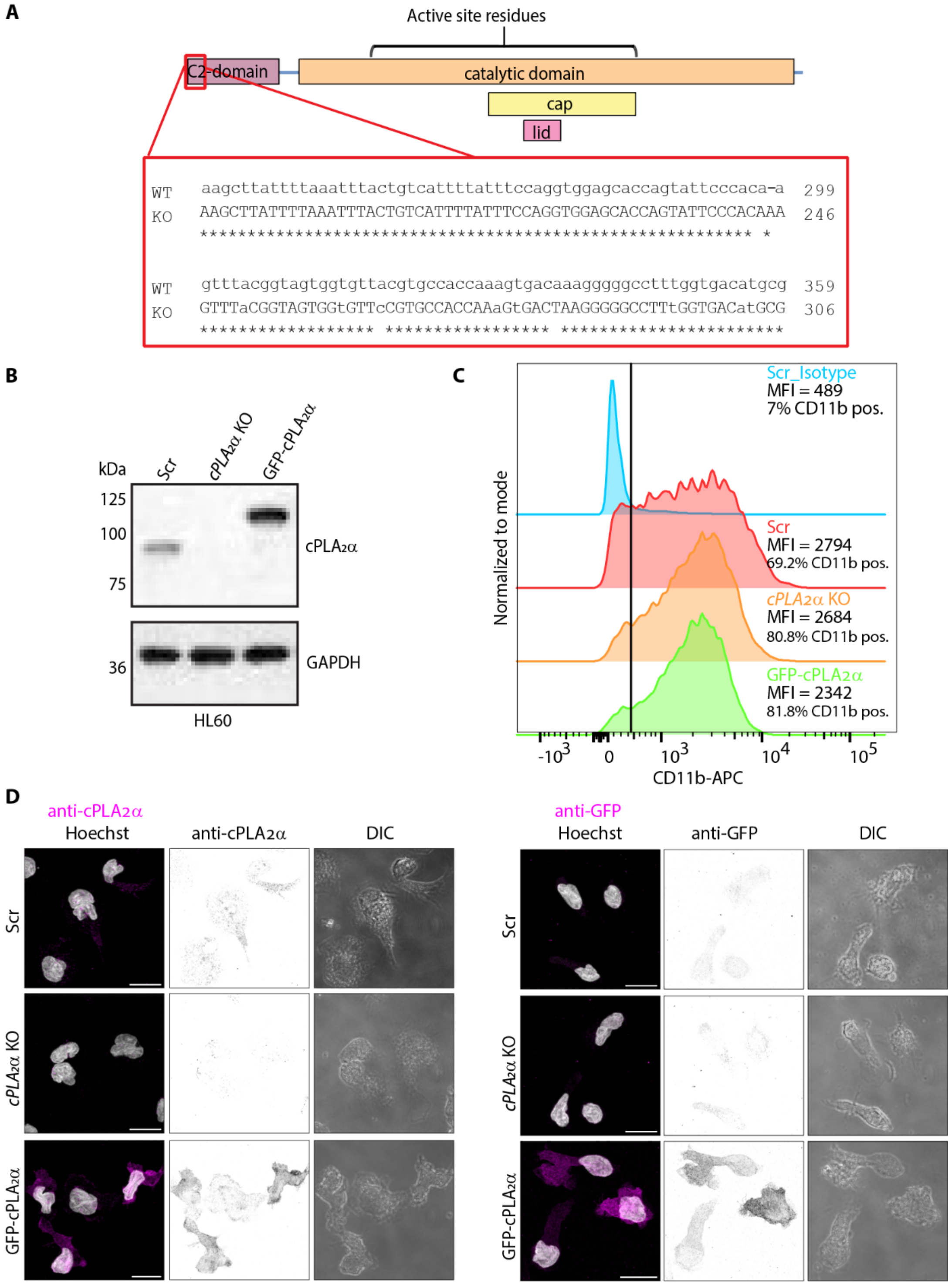

Fig. S1. Validation of cPLA₂α antibody and genetic manipulation.
(A) Schematic showing the mutations introduced at the beginning of the cPLA₂α C2-domain using the CRISPR-cas9 approach.
(B) Immunoblot of HL60 cells showing the level of cPLA₂α expression in KO and rescue cells.

(C) Histogram showing the percent CD11b-positive Scr, *cPLA$_2$α* KO, and GFP-cPLA$_2$α dHL60 cells, and levels of surface CD11b.

(D) Representative DIC and immunofluorescence microscopy images of Scr, *cPLA$_2$α* KO, and GFP-cPLA$_2$α HL60 (undifferentiated) immunostained with an antibody against cPLA$_2$α (left) and GFP (right). The scale is 5 μm.

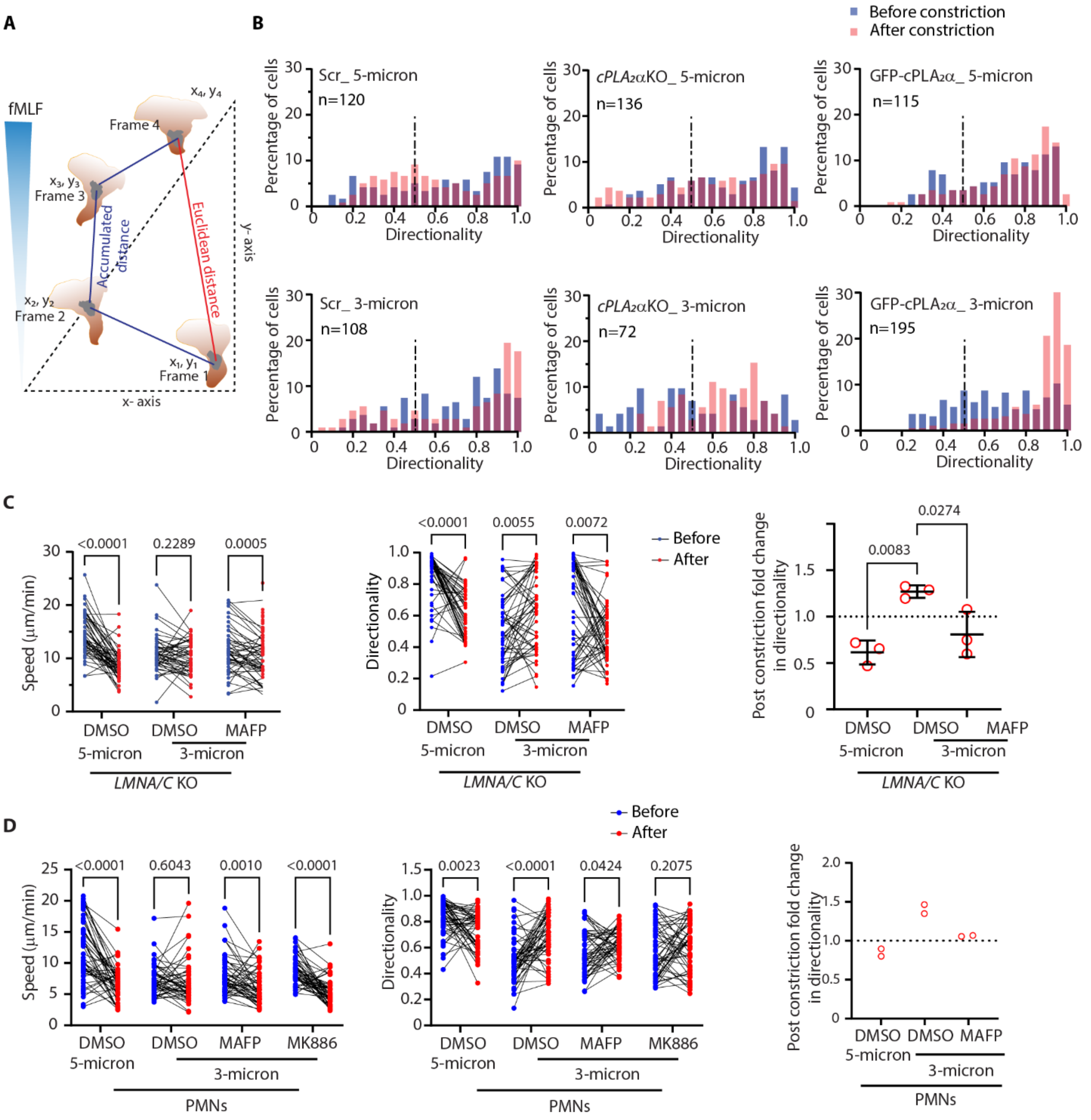

Fig. S2. Effect of LMNA/C on post-constriction persistence and pMLCII polarization.

(A) Schematic illustrating directional persistence and speed analysis based on accumulated versus Euclidean distance during chemotaxis toward fMLF.

(B) Bar graph showing directionality distributions of Scr, *cPLA₂α* KO, and GFP-cPLA₂α dHL60 neutrophils migrating through 5-micron or 3-micron constrictions. Datapoints (n) pooled from three independent experiments were used for frequency distribution analysis.

(C) Before-after line graph of migration speed and directionality of *LMNA/C* KO cells before and after passage through 3- or 5-micron constrictions in the presence of DMSO or MAFP, showing 50 randomized datapoints (circles) obtained from three independent experiments plotted. The *P* values calculated using multiple paired t-test were presented. Scatter plot (right) of median fold change in directionality presented as mean ± SD, with each circle representing an independent experiment.

(D) Before-after line graph of migration speed and directionality of PMNs before **and** after passage through 3- or 5-micron constrictions in the presence of DMSO, MAFP, or MK886, showing 50 randomized datapoints (circles) obtained from two independent experiments plotted. The *P* values calculated using multiple paired t-test were presented. Scatter dot plot of median fold change in directionality presented as mean ± SD, with each circle representing an independent experiment.

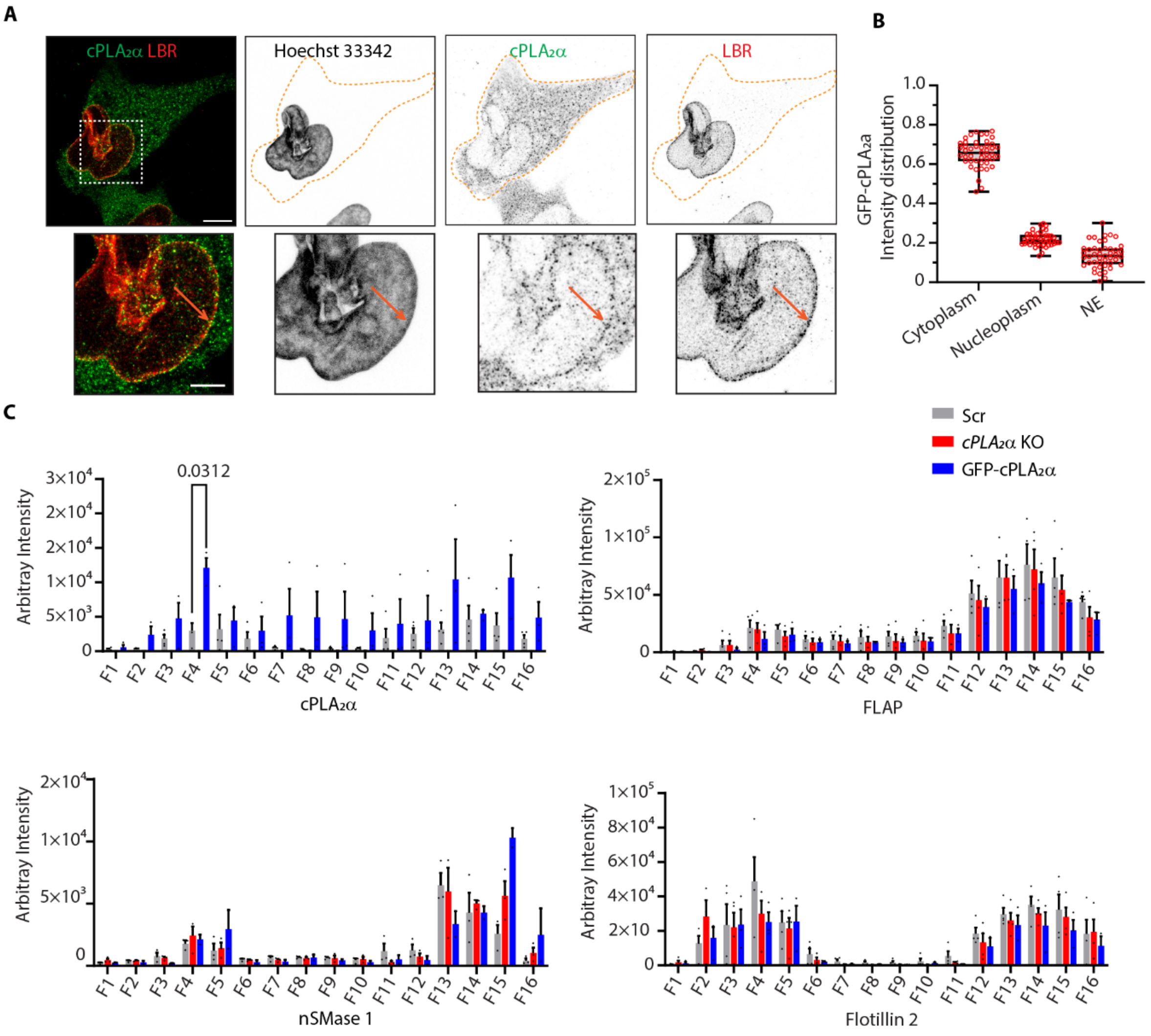

Fig. S3. NE distribution of GFP-cPLA$_2$α in dHL60 neutrophils. (A-B) Four-fold expansion microscopy images (A) of dHL60 neutrophils stably expressing GFP-cPLA$_2$α chemotaxing towards fMLF, fixed and stained with anti-GFP antibody and LBR, and quantified (B) for cytoplasm, nucleoplasm, and NE distribution. Scale is 5 µm, and 2 µm in the zoomed inset. Datapoints (45, red circles) from three independent experiments are plotted as mean ± s.e.m., and *P* values determined using ordinary one-way ANOVA are shown. **(C)** Graphs showing the cPLA$_2$α, FLAP, nSMase 1, and Flotillin 2 distribution in DRM and DSM fractions of NE obtained from activated dHL60 neutrophils. Data are plotted as mean ± s.e.m. of four independent experiments. *P* values determined using ratio-paired t-test are shown.

2. Movie legends

Movie S1: 3D volumetric view of the dHL60 neutrophils migrating under agarose towards fMLF, fixed and stained with phalloidin (red) and LBR (green). Representative of N=3.

Movie S2: 3D volumetric view of the fMLF-activated dHL60 neutrophils plated over aligned microfibers (red), fixed and stained for phalloidin (green) and Hoechst 33342 (blue). Representative of N=3.

Movie S3: Chemotaxis of GFP-$cPLA_2\alpha$ expressing and *cPLA₂α* KO dHL60 neutrophils, stained with Hoechst 33342 (magenta), migrating through 3-micron constriction C^3. Frame rate is 3 fps. Representative of N=4.

Movie S4: 3D volumetric view of the isolated nuclei showing the increased association of GFP-$cPLA_2\alpha$ with ceramide at the NE upon neutrophil activation. Representative of N=3.
